# Group B streptococcus infection during pregnancy and infancy: estimates of regional and global burden

**DOI:** 10.1016/S2214-109X(22)00093-6

**Published:** 2022-04-28

**Authors:** Bronner P Gonçalves, Simon R Procter, Proma Paul, Jaya Chandna, Alexandra Lewin, Farah Seedat, Artemis Koukounari, Ziyaad Dangor, Shannon Leahy, Sridhar Santhanam, Hima B John, Justina Bramugy, Azucena Bardají, Amina Abubakar, Carophine Nasambu, Romina Libster, Clara Sánchez Yanotti, Erzsébet Horváth-Puhó, Henrik T Sørensen, Diederik van de Beek, Merijn W Bijlsma, William M Gardner, Nicholas Kassebaum, Caroline Trotter, Quique Bassat, Shabir A Madhi, Philipp Lambach, Mark Jit, Joy E Lawn, GBS Danish and Dutch collaborative group for long term outcomes, GBS Low and Middle Income Countries collaborative group for long term outcomes, GBS Scientific Advisory Group, epidemiological sub-group, CHAMPS team

**Affiliations:** a Department of Infectious Disease Epidemiology, London School of Hygiene & Tropical Medicine, London, UK; b Maternal, Adolescent, Reproductive & Child Health Centre, London School of Hygiene & Tropical Medicine, London, UK; c Department of Medical Statistics, London School of Hygiene & Tropical Medicine, London, UK; d South African Medical Research Council, Vaccines and Infectious Diseases Analytical Unit, Faculty of Health Sciences, University of the Witwatersrand, Johannesburg, South Africa; e Department of Paediatrics and Child Health, Faculty of Health Sciences, University of the Witwatersrand, Johannesburg, South Africa; f Neonatology Department, Christian Medical College, Vellore, India; g Centro de Investigação em Saúde de Manhiça, Maputo, Mozambique; h ISGlobal, Hospital Clínic, Universitat de Barcelona, Barcelona, Spain; i Neuroscience Research Group, Department of Clinical Sciences, Kenyan Medical Research Institute, Wellcome Trust, Kilifi, Kenya; j Institute for Human Development, Aga Khan University, Nairobi, Kenya; k Fundación INFANT, Buenos Aires, Argentina; l Department of Clinical Epidemiology, Aarhus University, Aarhus, Denmark; m Department of Neurology, Amsterdam Neuroscience, Amsterdam UMC, University of Amsterdam, Amsterdam, Netherlands; n Department of Paediatrics, Amsterdam UMC, University of Amsterdam, Amsterdam, Netherlands; o Institute for Health Metrics and Evaluation, University of Washington, Seattle, WA, USA; p Departments of Global Health and Health Metrics Sciences, University of Washington, Seattle, WA, USA; q Department of Anesthesiology and Pain Medicine, University of Washington, Seattle, WA, USA; r Disease Dynamics Unit, Department of Veterinary Medicine, University of Cambridge, Cambridge, UK; s Catalan Institution for Research and Advanced Studies, Barcelona, Spain; t Pediatrics Department, Hospital Sant Joan de Déu, University of Barcelona, Barcelona, Spain; u Consorcio de Investigación Biomédica en Red de Epidemiología y Salud Pública, Madrid, Spain; v Department of Science and Technology and National Research Foundation: Vaccine Preventable Diseases, Faculty of Health Sciences, University of the Witwatersrand, Johannesburg, South Africa; w Department of Immunization, Vaccines and Biologicals, WHO, Geneva, Switzerland

## Abstract

**Background:**

Group B streptococcus (GBS) colonisation during pregnancy can lead to invasive GBS disease (iGBS) in infants, including meningitis or sepsis, with a high mortality risk. Other outcomes include stillbirths, maternal infections, and prematurity. There are data gaps, notably regarding neurodevelopmental impairment (NDI), especially after iGBS sepsis, which have limited previous global estimates. In this study, we aimed to address this gap using newly available multicountry datasets.

**Methods:**

We collated and meta-analysed summary data, primarily identified in a series of systematic reviews published in 2017 but also from recent studies on NDI and stillbirths, using Bayesian hierarchical models, and estimated the burden for 183 countries in 2020 regarding: maternal GBS colonisation, iGBS cases and deaths in infants younger than 3 months, children surviving iGBS affected by NDI, and maternal iGBS cases. We analysed the proportion of stillbirths with GBS and applied this to the UN-estimated stillbirth risk per country. Excess preterm births associated with maternal GBS colonisation were calculated using meta-analysis and national preterm birth rates.

**Findings:**

Data from the seven systematic reviews, published in 2017, that informed the previous burden estimation (a total of 515 data points) were combined with new data (17 data points) from large multicountry studies on neurodevelopmental impairment (two studies) and stillbirths (one study). A posterior median of 19·7 million (95% posterior interval 17·9–21·9) pregnant women were estimated to have rectovaginal colonisation with GBS in 2020. 231 800 (114 100–455 000) early-onset and 162 200 (70 200–394 400) late-onset infant iGBS cases were estimated to have occurred. In an analysis assuming a higher case fatality rate in the absence of a skilled birth attendant, 91 900 (44 800–187 800) iGBS infant deaths were estimated; in an analysis without this assumption, 58 300 (26 500–125 800) infant deaths from iGBS were estimated. 37 100 children who recovered from iGBS (14 600–96 200) were predicted to develop moderate or severe NDI. 40 500 (21 500–66 200) maternal iGBS cases and 46 200 (20 300–111 300) GBS stillbirths were predicted in 2020. GBS colonisation was also estimated to be potentially associated with considerable numbers of preterm births.

**Interpretation:**

Our analysis provides a comprehensive assessment of the pregnancy-related GBS burden. The Bayesian approach enabled coherent propagation of uncertainty, which is considerable, notably regarding GBS-associated preterm births. Our findings on both the acute and long-term consequences of iGBS have public health implications for understanding the value of investment in maternal GBS immunisation and other preventive strategies.

**Funding:**

Bill & Melinda Gates Foundation.

## Introduction

Neonatal infections are a major contributor to the global burden of diseases, with an estimated 6·9 million incident cases of presumed severe neonatal infections each year in low-income and middle-income countries alone, causing approximately half a million deaths worldwide in 2012.^1^ One of the leading pathogens causing these infections is group B streptococcus (GBS),^2^, ^3^ which has been recognised for more than 5 decades^4^, ^5^ as causing invasive GBS (iGBS) disease in young infants, with a high case fatality rate. A systematic review, published in 2012, described the iGBS incidence in infants for several regions,^6^ and the first set of global estimates for GBS,^3^, ^7^ including maternal GBS disease,^8^ stillbirths due to GBS,^9^ and an association between preterm birth and GBS colonisation,^10^ were published in 2017 using data for 2015. In the context of the UN call for expanding effective preventive interventions targeting the main causes of neonatal mortality,^11^ an updated and comprehensive estimation of the full GBS burden is necessary to guide and accelerate the development of improved preventive strategies, including maternal GBS vaccines.^12^, ^13^

**Research in context**

**Evidence before this study**

A previous study by Seale and colleagues estimated the global group B streptococcus (GBS) burden, including several outcomes that affect public health. The study quantified that GBS affected 21·7 million pregnant women globally in the year 2015 and, by applying a compartmental model with sequential risks, it predicted 319 000 cases of invasive GBS (iGBS) disease and 90 000 (95% uncertainty range, 36 000–169 000) deaths during early infancy. That study highlighted major data gaps, notably regarding neurodevelopmental impairment (NDI) in children who recovered from iGBS. Preterm birth associated with GBS was not estimated. We searched PubMed for studies on the global burden of GBS, published in English, up to Aug 1, 2021, using the search terms (Group B Streptococcus OR Streptococcus agalactiae) AND (global or worldwide or international) AND (burden OR incidence OR mortality OR morbidity OR burden OR prevalence OR survival) and found no other relevant papers. We also searched for data on GBS-related stillbirths and NDI after iGBS up to Feb 1, 2021, and included newly available published and unpublished data on these outcomes.

**Added value of this study**

Our new estimates for 2020 include a synthesis of data for each GBS outcome, including published and unpublished data, with specifically designed data collection on long-term NDI outcomes in both low-income and high-income settings. Our study included the largest data input yet on GBS, to the best of our knowledge, suppporting some of the findings of the previous study, notably the high number of women with GBS colonisation (∼20 million), infant iGBS cases (231 000 early-onset and 162 000 late-onset cases), and deaths caused by early infancy iGBS in every region (91 000), with a disproportionate burden in sub-Saharan Africa. Our findings for these outcomes are similar to those from the last study, despite differences in statistical approaches. Notably, we also estimated the risk of NDI after iGBS sepsis, not just after meningitis, based on new data on NDI among patients with iGBS from an electronic cohort in Denmark and five low-income and middle-income countries (Argentina, India, Kenya, Mozambique, and South Africa), which increased the estimated annual number of patients recovering from moderate or severe NDI. The estimated number of stillbirths due to GBS is notable (46 000) and crucial to count. We also estimated, for the first time, the potential preterm burden associated with GBS. Although this is potentially substantial, at more than 0·5 million cases, there is wide uncertainty, and it still represents a small proportion (3·5%) of the overall preterm birth burden, at 15 million births per year.

**Implications of all the available evidence**

Our findings show a higher burden of iGBS than previously estimated, primarily because of the previously unquantified number of NDI cases after GBS sepsis. We note that, even in regions with good coverage of intrapartum antibiotic prophylaxis, antibiotic prophylaxis, usually given around the time of birth, is unlikely to prevent most stillbirths, GBS-associated preterm birth, or late-onset iGBS, which is more likely to present as meningitis and has a high risk of NDI. Notably, a high proportion of the burden is in low-income and middle-income countries, where intrapartum antibiotic administration is more challenging to implement. Data gaps in this area that are a priority require more ambitious studies; for example, multicountry pregnancy cohort studies from the first trimester with more frequent testing of GBS colonisation could improve data for GBS-associated preterm risk as well as GBS stillbirths and maternal sepsis, which have been understudied in all settings. Our results will enable the first global cost-effectiveness analysis for maternal GBS vaccination and provide an imperative for more rapid progress given several decades of vaccine development so far.

A notable gap identified in earlier estimates of the GBS-related burden of disease was the associated risk of neurodevelopmental impairment (NDI), with no data available to estimate the risk of NDI after iGBS sepsis, and few data on NDI after iGBS meningitis.^14^ In 2021, data on GBS sepsis, a more frequent clinical presentation of this infection compared with meningitis, have been published: a large cohort study in Denmark and the Netherlands showed that GBS sepsis also leads to an increase in NDI risk and special education needs.^15^ In addition to these direct consequences of invasive infections, maternal GBS colonisation has also been associated with preterm births.^10^, ^16^ An association between GBS colonisation and preterm birth should be considered, given that prematurity causes deaths and disability in every region.^17^

In this study, we aimed to advance the estimates of the GBS burden from the year 2015^7^ by updating the estimated burden for 2020, including more input data from novel studies, and applying a different statistical approach to estimate, for 183 countries, the numbers of: (1) cases of maternal colonisation, (2) iGBS cases in the first 3 months of life, (3) deaths among infants with iGBS, (4) patients who recovered from iGBS with NDI, (5) stillbirths caused by GBS, (6) cases of maternal iGBS, and (7) excess preterm births associated with maternal GBS colonisation. Throughout the manuscript we highlight key data gaps, explain assumptions, and discuss the limitations of our approach, in line with recommended estimation practices.^18^

## Methods

### Overview

**Figure 1:**
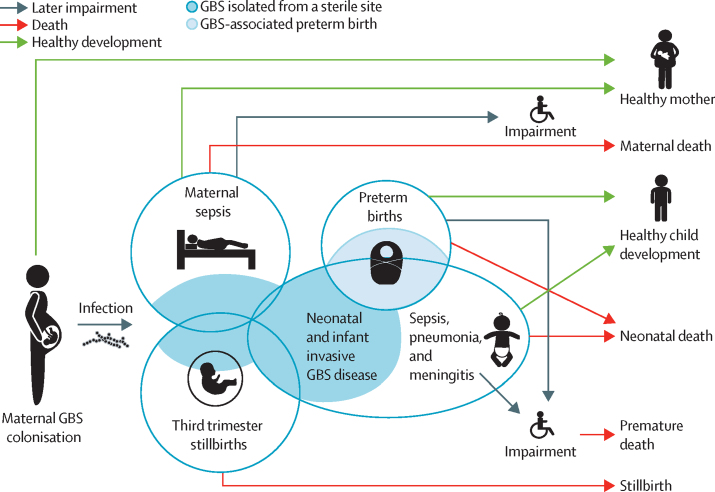
Overview of GBS colonisation in pregnancy and relevant outcomes During pregnancy, GBS can cause stillbirths, be associated with prematurity, and lead to maternal infection or, more rarely, death. Some newborn babies born to mothers who are GBS colonised develop invasive disease during the first week of life. Infants can also develop GBS disease between days 7 and 89. The fatality risk is high, and depends on adequate access to care. Of those children who survive the acute episode, some will be diagnosed with neurodevelopmental impairment. Neurodevelopmental impairment risks after GBS meningitis and GBS sepsis differ and were modelled separately. This figure was adapted from a study by Lawn and colleagues, with permission.^3^ GBS=group B streptococcus.

We collated aggregated data for all outcomes (cases of maternal colonisation, iGBS cases in the first 3 months of life, deaths among infants younger than 3 months with iGBS, patients who recovered from iGBS with NDI, stillbirths caused by GBS, cases of maternal iGBS, and excess preterm births associated with maternal GBS colonisation). Most studies included were identified in a series of systematic reviews published in 2017,^3^, ^7^, ^8^, ^9^, ^10^, ^14^, ^19^, ^20^, ^21^ and we included new data on stillbirths and NDI. Note that the search strategy, selection criteria, data extraction, and assessment of study quality were described in the original publications of these systematic reviews. Bayesian hierarchical models^22^ were used to analyse the evidence. We estimated the absolute burden for 2020 on the basis of the country-specific numbers of births in the UN World Population Prospects 2019 for 183 countries included in the dataset based on population size (only countries with 90 000 or more inhabitants were listed). All relevant outcomes were studied as shown in figure 1 and panel 1, with predefined case definitions. Bayesian models allowed a coherent propagation of uncertainty in the estimation of the burden and accounted for the multilevel structure of the data (eg, for several outcomes, multiple studies were identified per region). Posterior medians and intervals (95% posterior intervals) were reported that account for the uncertainty in the parameter estimation; relevant sensitivity analyses were also reported. Parameters were estimated either at the country (eg, maternal colonisation), regional (eg, case fatality rate), or global (eg, risk of NDI after GBS meningitis) levels, depending on data availability (appendix pp 4–22). Details on the statistical models, including assumptions and prior probability distributions, are in the appendix (pp 4–22). Our estimation process followed the GATHER statement.^18^ Results are shown by Sustainable Development Goals regions.

Panel 1 **Case definitions used for estimates3,7–10,14,19–21,23**

• Group B streptococcus (GBS) maternal colonisation: isolation by culture of GBS from either the vagina (high or low), rectum, or peri-anal region at any time during pregnancy
• Maternal GBS disease: laboratory isolation of GBS from sterile site in pregnant or post-partum women (up to 42 days post partum), with clinical signs of sepsis
• Stillbirth GBS invasive disease: birth of a fetus weighing >1000 g or ≥28 weeks' gestational age, or both, with no signs of life and evidence of GBS invasive disease from a normally sterile site such as foetal blood, lung aspirate, or cerebrospinal fluid
• Neonatal and infant GBS invasive disease: laboratory isolation of *Streptococcus agalactiae* from a normally sterile site in an infant aged 0–89 days with signs of clinical disease, including meningitis, sepsis, or bacteraemic pneumonia
• Neurodevelopment impairment in children after GBS invasive disease: cognitive or motor, vision, or hearing, or a combination, impairment in patients who have recovered from invasive infant GBS disease isolated from a normally sterile site
• Preterm birth associated with GBS maternal colonisation: delivery before completion of 37 weeks of gestation from a mother with maternal GBS colonisation isolated from vaginal, cervical, or rectal swabs, or a combination

### Maternal GBS colonisation

GBS colonisation of the maternal genitourinary tract is associated with early-onset iGBS (EOGBS)^24^, ^25^ and quantification of its frequency is a key step in the estimation process. We included data from a systematic review,^23^ and developed a hierarchical model to regress country-level maternal GBS colonisation prevalence on country-level covariates.^26^ Using this approach, we estimated the colonisation prevalence in pregnant women for countries with and without GBS colonisation data, adjusting for study differences (appendix pp 4–5) in terms of the swab site and laboratory diagnostics, and accounting for parameter uncertainty.

### EOGBS and late-onset iGBS (LOGBS)

iGBS in infants might present as EOGBS (0–6 days) or LOGBS (7–89 days), and clinical syndromes include sepsis or meningitis (figure 1, panel 1). Given that routine surveillance data might underestimate EOGBS in most settings, as argued previously by Edmond and colleagues^6^ and Dagnew and colleagues,^27^ here we estimated the risk of EOGBS in neonates born to mothers who were GBS colonised. For modelling EOGBS risk, it was essential to consider intrapartum antibiotic prophylaxis use; hence we developed a model to regress study-level EOGBS risk in babies of mothers who were GBS colonised who received intrapartum antibiotic prophylaxis.^2^, ^19^ We used this regression in combination with national coverage data on intrapartum antibiotic prophylaxis, based primarily on a review with data for 92 countries,^20^ plus additional assumptions for those countries without data (appendix pp 6–7): we assumed 0% intrapartum antibiotic prophylaxis coverage in low-income countries and 80% coverage in high-income countries with no coverage data (appendix pp 6–7). In a secondary analysis, we used a Bayesian evidence synthesis model^26^ to combine data from studies directly assessing EOGBS incidence and studies estimating the risk of EOGBS among infants of mothers who were GBS colonised.

Similar to EOGBS, cases of LOGBS are likely to be under-reported in surveillance systems. Hence, we used an approach similar to a previous analysis^21^ and estimated region-specific proportions of LOGBS compared with EOGBS, which provided a multiplication factor to apply to EOGBS estimates. Full details are in the appendix (pp 6–9).

### Deaths among infants with iGBS

Since most EOGBS cases (68%) occur within 24 h of birth,^21^ we assumed a high case fatality rate of 90%, using similar assumptions to Seale and colleagues,^7^ for EOGBS cases without skilled birth attendance (a marker of low access to any health care, including antibiotic treatment). A dataset from UNICEF and WHO on country-level skilled birth attendance was used.^28^ For cases of patients with EOGBS with skilled birth attendance and all cases of LOGBS, we applied case fatality rates estimated by region on the basis of published literature (appendix pp 10–12). Assuming a fixed high mortality rate (of 90%) for cases of EOGBS without skilled birth attendance means that uncertainty in this parameter, which is key in the estimation of number of deaths, is not incorporated in the estimates. We also undertook a sensitivity analysis that considered the scenario that EOGBS without skilled birth attendance could have the same regional case fatality rate as EOGBS with skilled birth attendance.

### Long-term moderate or severe NDI among patients who recovered from iGBS

iGBS can lead to NDI in those who recover. In this analysis, we estimated the risk of moderate and severe impairments, which is more likely to be consistent across settings and study designs compared with milder NDI types; in the appendix (pp 15–16), we present the results of analyses that also considered mild NDIs. For meningitis, we used studies reviewed in 2017,^14^ novel data from a national cohort in Denmark (which included children with outcome data available at the age of 10 years),^15^ and reanalysis of data from a multicentre observational study^29^ performed in Argentina, India, Mozambique, Kenya, and South Africa including all meningitis cases, irrespective of age (4–17 participants in each centre, with a total of 38 participants); that study was performed as part of this research project. For GBS sepsis, we used data from the same study in Denmark (805 children with data at the age of 10 years) and four smaller studies (4–61 participants in each study, with a total of 109 participants), also reviewed in a systematic review by Kohli-Lynch and colleagues,^14^ for high-income countries; for low-income and middle-income countries, data from the aforementioned multicentre study were used (22–31 participants in each centre, with a total of 108 participants) in a separate meta-analysis. Note that data from the Argentinian site in the multicentre study were not included because the low proportion of identified patients who recovered from iGBS who were assessed might have been linked to selection bias. Definitions of NDI, including of moderate and severe presentations, are described in detail in these previous studies;^14^, ^15^, ^30^ both the Danish study and the multicentre study, done as part of this project, used multidomain definitions consistent with the Global Burden of Disease, Injuries, and Risk Factors Study.^31^ Since most of these studies did not include comparator groups, our analysis does not estimate the association between iGBS and NDI, but rather the risk of moderate and severe NDI in patients who recovered from iGBS. Hence, to estimate the number of NDI cases attributable to iGBS, we used the risk of moderate or severe NDI in children without a history of iGBS in the Danish study (2·1%) as a conservative estimate of the comparative risk. Although the Danish cohort is the largest to date, and included information into the second decade of life, it is probable that the counterfactual risk varies considerably within and between countries. Similar to the EOGBS case fatality rate in children with no access to skilled birth attendance, this counterfactual risk was assumed to be fixed; hence estimates do not incorporate uncertainty in this parameter. Another assumption is that the risk of NDI estimated in these analyses represents a lifelong risk, which is probably an underestimation, because most studies recruited children younger than 10 years.

### Stillbirths due to GBS

Data inputs included a literature review on the proportion of stillbirths with evidence of GBS infection, with six studies published after 2000, one of which also included data collected before that year.^9^ We updated the searches and identified no additional published studies, but were able to add unpublished data from the Child Health and Mortality Prevention Surveillance (CHAMPS) network^32^ including seven study sites in Africa and Asia, corresponding to a total of 509 stillbirths. In the CHAMPS network, a panel of local experts evaluated all data available for stillbirths enrolled with a post-mortem investigation including clinical information and collection and testing of tissue and non-tissue specimens to establish whether GBS was involved in the causal chain leading to each stillbirth. We applied a hierarchical model to combine data within regions. For regions with no studies, we used the model to predict the proportion. Region-specific proportions of stillbirths caused by GBS were then applied to country-specific stillbirth risk from the Global Health Observatory data repository. Sensitivity analyses with different prior assumptions were included in the appendix (p 42).

### Maternal iGBS

GBS also causes disease in pregnant women. The little research on this, all from high-income countries, was reviewed by Hall and colleagues.^8^ We used data reported in that review, together with those from a study in England,^33^ to estimate the risk of GBS-related morbidity during pregnancy or post partum (up to 42 days after delivery). Of note, the inclusion of the 2020 study in England was not the result of a systematic search but was suggested by the project's Scientific Advisory Group. Since these studies, which primarily reported cases where GBS was cultured from blood or cerebrospinal fluid, or both, did not estimate risk given maternal GBS carriage, we applied our estimates directly to country-specific number of births.

### Excess preterm births associated with maternal GBS colonisation

Using previously reported data,^10^ we estimated the association between maternal GBS colonisation and preterm births. To incorporate all available evidence,^34^ we performed a meta-analysis on case-control studies and used the posterior distribution of the coefficient as a prior distribution in a meta-analysis model of cohort and cross-sectional studies. The overall odds ratio was used, together with country-level frequencies of preterm births,^35^ to calculate the excess number of preterm births associated with maternal GBS colonisation. Two different approaches were used for this calculation, which also required estimated country-level prevalence of maternal GBS colonisation; in the appendix (pp 21–22), we present the results of the meta-analyses and describe these approaches.

### Bayesian models

All analyses were done using the Hamiltonian Monte Carlo algorithm in PyStan (version 2.19), the interface for the Stan libraries in Python (version 3.7).^36^ Details on the models are presented in the appendix (pp 4–22, 43), including additional assumptions in our analyses and probable limitations.

### Study ethics approval

For the primary data collection in high-income, low-income, and middle-income countries, the overarching protocol for the observational study was granted ethics approval at the London School of Hygiene & Tropical Medicine (approval number 16246). Institutional review boards in each of the countries granted ethics approval (Argentina approval number protocol EGB-1; India approval numbers 11723 [Christian Medical College Vellore], 2019–7034 [Indian Council of Medical Research]; Kenya approval number SERU/CGMR-C/164/3882; Mozambique approval number 98/CNBS/2019; and South Africa approval number M190241), as well as the institutional review board of WHO (approval number ERC.0003169). The Danish electronic cohort study was approved by the Danish Data Protection Agency (record number 2015–57–0002). In the Netherlands, the study protocol (EPI-408) was submitted to the Centre for Clinical Expertise at the National Institute for Public Health and the Environment. The study protocol was exempted from further approval by an ethics research committee, according to Dutch law for medical research involving human patients. This study was reviewed by the Centers for Disease Control and Prevention and was conducted consistent with applicable federal law and Centers for Disease Control and Prevention policy.

### Role of the funding source

The funder of the study had no role in study design, data collection, data analysis, data interpretation, or writing of the report.

## Results

Input data per parameter are summarised in table 1. Maternal GBS colonisation had the most data, in terms of number of studies, and subsequent variables have fewer inputs, notably maternal infection and stillbirths. Some regions are markedly under-represented; for example, only one study was identified on GBS diagnosis in stillbirths in Asia (Bangladesh), with none from Asia for maternal infection. Most studies assessing the risk of EOGBS in babies born to mothers who were GBS colonised and all studies on GBS-related maternal morbidity were from high-income countries.

**Table 1:** Summary of the input data for the estimation for each parameter relevant to GBS burden in pregnancy and infancy

	**Parameter(s) modelled**	**Data included**	**Input data overview**	**Level of estimation**
Maternal GBS colonisation	Prevalence	325 studies from 82 countries^23^	Number of studies per country: median, 2 (range, 1–31); number of study participants: 349 (35–17 430); number of mothers who are GBS colonised: 48 (1–2911); and publication date range: 1981–2016	Country
EOGBS risk	Risk of EOGBS in babies born to mothers who were GBS colonised (regression used to measure this risk using intrapartum antibiotic prophylaxis covariate)	28 studies assessing the risk of EOGBS in babies born to mothers who were GBS colonised^19^	Number of studies done in high-income countries: 23; number of mothers who are GBS colonised per study: median, 450 (range, 216–3819); number of EOGBS cases in each study: 2 (0–24); publication date range: 1979–2016	Country
LOGBS risk	Proportion of all iGBS cases that are LOGBS	20 studies that directly assess both EOGBS and LOGBS incidence^21^	Number of studies per region: range, 1–12; number of GBS cases per study: median, 114 (range, 15–856); publication date range: 2002–16	Region
Death among infants with iGBS	Case fatality rates for EOGBS and LOGBS cases	47 studies were used to estimate the case fatality rate for EOGBS cases and 29 studies were used to estimate the case fatality rate for LOGBS cases^21^	Number of studies per region: EOGBS range, 7–19, and LOGBS range, 3–14; number of GBS cases per study: EOGBS median, 21 (range, 1 − 517), and LOGBS median, 43 (range, 3–373); publication date range: 2002–16	Region
Patients who recovered from iGBS (meningitis) with NDI	Risk of moderate or severe NDI in those who recovered from iGBS (meningitis)	20 studies^14^	Number of study participants: range, 4–103; number of children with moderate or severe NDI: range, 0–30; publication date range: 1982–2022	Global
Patients who recovered from iGBS (sepsis) with NDI	Risk of moderate or severe NDI in those who recovered from iGBS (sepsis)	Nine studies (five in high-income countries and four in low-income and middle-income countries)^14^, ^15^	Number of study participants: median, 36 (range, 4–805) in high-income countries; range, 22–31 in low-income and middle-income countries; number of children with moderate or severe NDI: range, 0–31; publication date range: 1974–2022	Region
Stillbirths due to GBS	Proportion of stillbirths with evidence of GBS infection	Data from six studies done after 2000, in addition to data from the CHAMPS network on seven local studies (ten countries in total)^9^, ^32^	Number of studies done in Africa: nine; number of studies done in Asia (Bangladesh): one; number of stillbirths investigated: median, 80 (range, 18–5175); number of GBS-related stillbirths: 2 (0–37); publication date range: 2001–17 (and unpublished data)	Region
Maternal iGBS	Risk of GBS-related maternal disease	Five studies^8^, ^33^	Number of study participants: median, 150 043 (range, 59 491–1 327 556); number of maternal cases: 57 (8–493); publication date range: 2013–20	Global
Excess preterm births associated with maternal GBS colonisation	Odds ratio of the association between preterm births and maternal GBS colonisation	Nine case-control studies and 28 cohort and cross-sectional studies^10^	Number of study participants: case-control studies median, 206 (range, 82–329), and cohort or cross-sectional studies: 998 (62–216 132); number of preterm births: case-control studies 84 (37–151), and cohort or cross-sectional studies 90 (3–17 018); publication date range: 1982–2016	Global

For more details of input data see the appendix. EOGBS=early-onset iGBS. GBS=group B streptococcus. iGBS=invasive GBS. LOGBS=late-onset iGBS. NDI=neurodevelopmental impairment.

**Figure 2:**
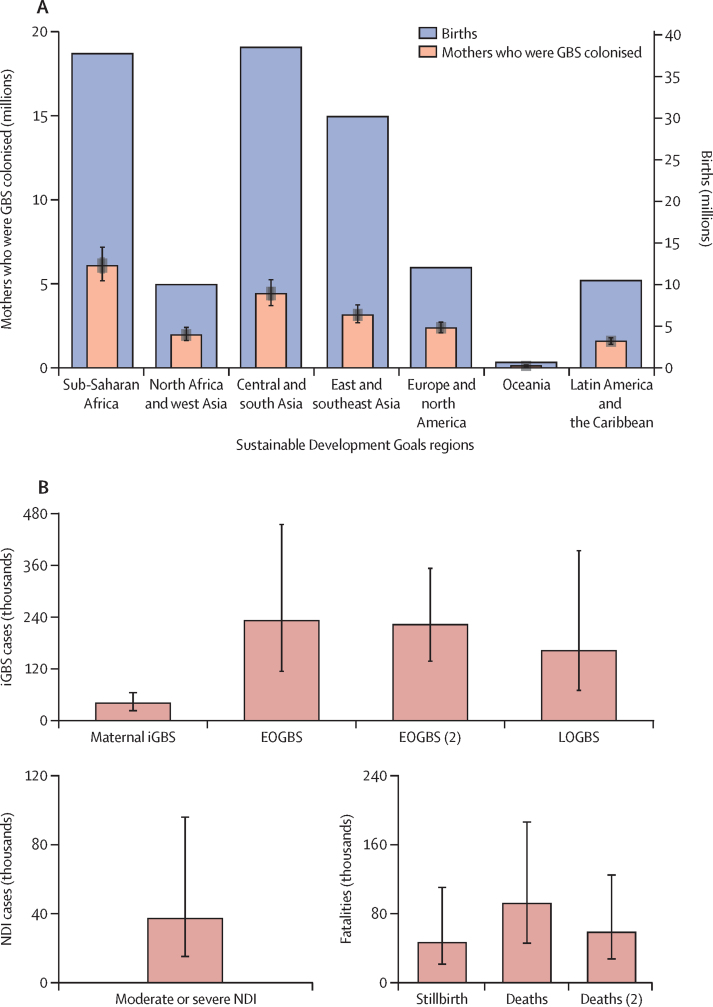
Global burden of outcomes related to GBS in pregnancy and infancy (A) Number of pregnant women who were GBS colonised by Sustainable Development Goal regions. The height of the orange bars represents the median, and 2·5–97·5%, 25–70%, and 40–60% percentile intervals are presented by error bars with different widths. The blue bars correspond to the total number of births in each region. (B) Estimated global burden of GBS cases, deaths, and NDIs. In the top panel, global numbers (posterior median and 95% percentile interval) of patients with iGBS (maternal, EOGBS, and LOGBS) are shown. The bottom left plot presents the number of children estimated to develop moderate and severe NDI after iGBS in 2020; and the bottom right panel shows the estimated numbers of stillbirths and deaths in infants with iGBS in 2020. In the top panel, EOGBS (2) corresponds to estimates that included both direct and indirect data on incidence; and in the bottom right panel, deaths (2) corresponds to the sensitivity analysis that did not assume a higher mortality in EOGBS cases in the absence of skilled birth attendance. EOGBS=early-onset iGBS. GBS=group B streptococcus. iGBS=invasive GBS. LOGBS=late-onset iGBS. NDI=neurodevelopmental impairment.

**Figure 3:**
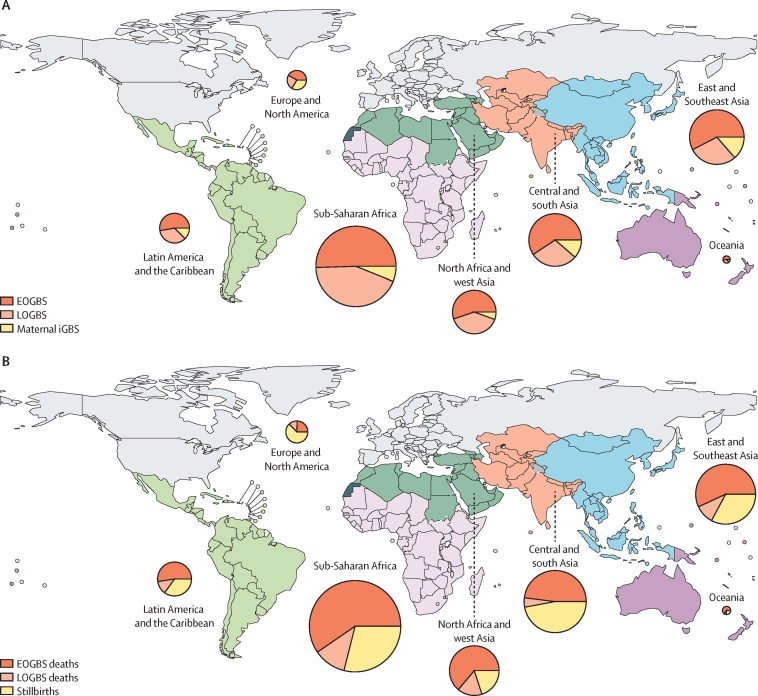
Region-specific relative distribution of GBS burden (A) Cases of EOGBS and LOGBS as well as maternal iGBS. (B) Deaths during EOGBS or LOGBS and stillbirths. The map is coloured showing Sustainable Development Goal regions. The areas of the pie charts are proportional to region-specific numbers. The pie charts present the proportions of cases in different regions that affect babies and women. Posterior medians were used for each of the outcomes; uncertainty in the proportions is therefore not presented. EOGBS=early-onset iGBS. GBS=group B streptococcus*.* iGBS=invasive GBS. LOGBS=late-onset iGBS.

19·7 million (95% posterior interval, 17·9–21·9) pregnant women were estimated to be colonised with GBS globally in 2020, with the highest numbers in sub-Saharan Africa (6·1 million [5·2–7·2]) and central and south Asia (4·4 million [3·7–5·2]; figure 2A; appendix pp 35–36). After accounting for intrapartum antibiotic prophylaxis policy coverage,^7^, ^20^ we estimated 231 800 (114 100–455 000) neonates developed EOGBS, with 90 800 (43 000–186 600) cases estimated to occur in sub-Saharan Africa, and 4300 (2000–7600) in Europe and north America (table 2). In a secondary analysis for the same countries, which included both direct and indirect evidence on incidence, the estimated number of global EOGBS cases was 222 500 (138 000–353 300; appendix p 37). Using region-specific proportions of iGBS cases presenting as LOGBS (appendix p 38), we estimated that 162 200 (70 200–394 400) infants developed LOGBS (table 2). Figure 3A shows the relative distribution of infant and maternal GBS cases by region. We present the combined incidence of EOGBS and LOGBS cases in different regions in the appendix (p 24).

**Table 2:** Sustainable Development Goal region estimates of acute and long-term outcomes

	**Stillbirths**	**EOGBS**	**LOGBS**	**Infant deaths after iGBS (both EOGBS and LOGBS)**
Sub-Saharan Africa	20 300 (9000–40 500)	90 800 (43 000–186 600)	78 100 (30 000–218 700)	50 600 (23 800–108 400)
North Africa and west Asia	2300 (1000–5800)	29 000 (13 800–58 700)	20 800 (8400–52 800)	9600 (4300–20 800)
Central and south Asia	14 700 (3600–51 500)	47 300 (24 300–89 900)	23 600 (6100–68 600)	16 700 (8200–33 500)
East and southeast Asia	4600 (1100–16 200)	45 700 (21 600–92 900)	22 600 (5700–68 200)	9700 (4200–22 600)
Latin America and the Caribbean	1800 (300–11 700)	12 800 (6700–24 400)	8400 (2700–29 200)	3600 (1600–8200)
Oceania	100 (20–600)	700 (300–1500)	400 (100–2600)	300 (100–900)
Europe and North America	700 (200–1800)	4300 (2000–7600)	2500 (1000–5300)	400 (200–800)
Global	46 200 (20 300–111 300)	231 800 (114 100–455 000)	162 200 (70 200–394 400)	91 900 (44 800–187 800)

Data shown as posterior medians (95% posterior intervals) of GBS-related stillbirths, EOGBS cases, LOGBS cases, and infant deaths during iGBS in 2020 by region. The last two digits in each number were rounded down, except for numbers less than 100, as done in previous estimates. EOGBS=early-onset iGBS. GBS=group B streptococcus. iGBS=invasive GBS. LOGBS=late-onset iGBS.

Assuming a higher case fatality rate in the absence of a skilled birth attendant, an estimated 91 900 (44 800–187 800) deaths occurred in infants with either EOGBS or LOGBS globally (Figure 2, Figure 3; table 2; appendix p 39). In the appendix (p 40), we present a sensitivity analysis without this assumption, whereby 58 300 (26 500–125 800) infants were estimated to die of iGBS in 2020.

Using the country-specific stillbirth estimates for the year 2019 by the UN and region-specific proportions of stillbirths caused by GBS infection (appendix pp 36, 41), we estimate that globally, 46 200 (20 300–111 300) stillbirths resulted from in utero GBS infection in 2020.

Of the patients who recovered from iGBS, 20·7% (16·1–25·6) of those with GBS meningitis (all countries combined), 3·3% (1·0–7·6) with GBS sepsis in high-income countries, and 9·2% (2·4–22·8) with GBS sepsis in low-income and middle-income countries are predicted to develop moderate or severe NDI, which amounts to 37 100 children (14 600–96 200; figure 2B; panel 2). Using the risk of moderate and severe NDI in children with no history of iGBS^15^ as a counterfactual, we also crudely estimated the excess number of children with NDI due to iGBS, which was 30 400 (10 900–84 300; appendix pp 13–16).

Panel 2 **Estimated numbers of children with moderate and severe neurodevelopmental impairment after invasive group B streptococcus in 2020 by Sustainable Development Goal region**

• Sub-Saharan Africa, 15 000 (5400–42 300)
• North Africa and west Asia, 4900 (1800–13 200)
• Central and south Asia, 6600 (2500–17 900)
• East and southeast Asia, 6900 (2500–19 700)
• Latin America and the Caribbean, 2100 (800–6500)
• Oceania, 100 (30–400)
• Europe and North America, 400 (200–900)
• Global, 37 100 (14 600–96 200)

Data shown as posterior median and 95% posterior intervals. The last two digits in each number were rounded down, as done in previous estimates, except for numbers below 100.

The risk of GBS maternal disease was 0·29 (0·15–0·47) per 1000 deliveries and was assumed to be the same in all countries. Note that no studies in this analysis were done outside of Europe and North America. We applied this risk to the country-specific numbers of births to calculate the global number of maternal GBS cases: 40 500 (21 500–66 200; figure 2).

We estimated the meta-analytical odds ratio for the association between maternal GBS colonisation and prematurity to be 1·30 (1·02–1·71). We used country-specific preterm risk estimates and our estimates of maternal GBS colonisation prevalence to quantify the excess number of preterm births associated with GBS colonisation: 518 100 (36 900–1 142 300) globally, of which 172 300 (12 300–380 000) were in sub-Saharan Africa and 125 100 (8400–281 200) were in central and south Asia (table 3); which corresponded to 3·5% (0·2–7·7%) of preterm births globally.

**Table 3:** Estimated numbers of preterm births associated with maternal GBS colonisation in 2020 by Sustainable Development Goal region

	**Excess preterm births associated with GBS colonisation (%)**	**Excess preterm births associated with GBS colonisation (numbers)**
Sub-Saharan Africa	4·0 (0·3–8·9)	172 300 (12 300–380 000)
North Africa and west Asia	4·8 (0·4–10·6)	56 200 (4300–123 300)
Central and south Asia	2·6 (0·2–6·0)	125 100 (8400–281 200)
East and southeast Asia	2·7 (0·2–6·3)	68 500 (4900–157 200)
Latin America and the Caribbean	3·9 (0·2–8·6)	38 800 (2800–85 700)
Oceania	4·7 (0·3–10·6)	2800 (200–6400)
Europe and North America	5·1 (0·4–11·1)	52 700 (3800–114 400)
Global	3·5 (0·2–7·7)	518 100 (36 900–1 142 300)

Data shown as posterior medians (95% posterior intervals). The last two digits in each number were rounded down, except for numbers less than 100, as was done in previous estimates. GBS=group B streptococcus.

## Discussion

We present the most comprehensive estimates to date, to the best of our knowledge, of the global burden of GBS disease related to pregnancy. Our results suggest that the burden is even higher than previously quantified, in part because of novel data enabling the estimation of NDI cases after GBS sepsis and not just meningitis. It is likely that more than 200 000 infants developed EOGBS, and approximately 160 000 developed LOGBS in 2020. Globally, 91 900 deaths were estimated to have occurred in children with iGBS, with the highest numbers in sub-Saharan Africa and Asia. More data on the risk of moderate and severe NDI after GBS meningitis and novel findings on NDI after GBS sepsis allowed for a more comprehensive quantification of these outcomes. New data from Africa and Asia also allowed for a better estimation of stillbirths due to GBS.^37^ Finally, we estimated the potential annual burden of GBS-associated preterm births for the first time, at 0·5 million, with a wide uncertainty range. Altogether, these results highlight the large burden of GBS infection, better informing public health action, especially for prevention, and highlight a need for improved epidemiological data, including prospective multicentre pregnancy cohort studies in the highest burden regions, to reduce uncertainty in estimates.

Infants in sub-Saharan Africa have the highest burden of iGBS, with nearly half of all global GBS-related deaths occurring there, reflecting high colonisation rates, a near absence of intrapartum antibiotic prophylaxis policy adoption, and a mortality due to GBS of 23% (12–38%). Stillbirths caused by GBS are also high in sub-Saharan Africa, whereas in Asia there are few data available on GBS diagnosis in stillbirths. Of note, the stillbirth outcome was modelled as the proportion with evidence of GBS infection in a sterile site, and applied to the latest UN estimates by country for stillbirth rate. We note that the global estimated number of stillbirths has decreased from 2·6 million^38^ in 2015 to the current 2 million since we applied proportionate risk to the country-specific total numbers of stillbirths estimated by the UN for 2019, and the apparent decrease in GBS stillbirth estimates is partly because of lower UN stillbirth rate estimates. Given that maternal GBS colonisation presumably is a necessary step for GBS-related stillbirths, studies comparing the stillbirth risk in pregnant women with GBS colonisation versus pregnant women with no evidence of GBS colonisation, and that adjust for potential confounders, would probably improve the quantification of GBS stillbirth risk. Notably, our estimates highlight the importance of this public health problem, which in most settings is unrecognised partly because of the limited laboratory capacity to diagnose infections in stillbirths, but also because of the absence of an investigation of the causes of stillbirth, even in high-income settings.

NDI risk after GBS sepsis was a major data gap, and multicountry studies have allowed a more accurate estimation of the long-term consequences of iGBS than was feasible in the previous global estimate. On one hand, the risk of moderate or severe NDI after GBS sepsis is lower compared with the risk in children who had GBS meningitis, with the risk of NDI after GBS meningitis being similar to the risk after severe neonatal meningitis due to any bacterial pathogen.^39^ On the other hand, since sepsis is a more common presentation, our analysis suggests that the number of moderate or severe NDI cases is probably considerably higher than previously estimated. One limitation is that most studies on NDI after iGBS did not include a comparator group. As a crude approach to estimating the excess number of moderate or severe NDI cases due to GBS, we used the risk of NDI of children who had no history of iGBS in Denmark as a counterfactual risk. The risk was assumed to be fixed, ignoring the uncertainty in this parameter and probable variation that might occur in different settings. One meta-analysis of studies performed in low-income and middle-income countries,^40^ for example, estimated a lower prevalence of NDI in the general population in some settings, whereas other data, collected using sensitive tools for NDI assessment, suggest a higher baseline risk of moderate or severe NDI than the baseline risk estimated in the Denmark study (1–25% in studies with more than ten participants.^29^ Another limitation of our analysis is that different studies recruited children at different ages and used different NDI assessment tools or epidemiological designs, which might partly explain the substantial between-study variability in risk (appendix pp 29–32). Furthermore, unlike the previous estimates, we did not quantify neonatal encephalopathy with GBS to avoid double counting.^41^ Notably, NDI after iGBS will deeply affect children's lives, in particular those with moderate or severe impairment, as well as affect their parents, and will have economic consequences to society broadly. Our findings confirm the necessity of including support to and aftercare for those who recover from bacterial meningitis as one of the pillars of the Defeating Meningitis roadmap.^42^

Prematurity has been shown to be associated with GBS colonisation, and there are plausible mechanisms for this.^16^, ^43^ However, the most careful review to date highlighted the heterogeneity in estimates of this association from epidemiological studies, which might be related to differences between studies in terms of the timing of screening for GBS.^10^ Some cohort studies assessed the exposure of GBS colonisation late in the third trimester of pregnancy, hence missing preterm births occurring earlier in pregnancy and potentially underestimating the association. Given this challenge and the wide uncertainty range, although in our analysis we incorporated all the evidence available from observational studies, our estimates of the associated excess numbers need to be carefully interpreted. Although the number appears to be high, it is a small proportion (approximately 3·5%) of the approximately 15 million preterm births each year. We note that, in addition, preterm babies have a higher risk of iGBS, especially LOGBS,^44^ which we have not estimated.

Maternal iGBS was estimated at 0·29 per 1000 births, and a total of 40 500 cases per year, which might represent a considerable proportion of all iGBS cases in mothers and babies. However, all included studies were from high-income countries and, by using these data to represent all countries, we are likely to have underestimated the magnitude of this public health problem. Moreover, the few data on the fatality risk during these episodes^8^ prevented the estimation of maternal deaths, which might be higher where data are absent.

Incidence of EOGBS reflects both the prevalence of maternal GBS colonisation and the risk of disease in babies born to mothers who are GBS colonised. By inferring case numbers from these two sources, we attempted to avoid the underestimation consistently described for studies and routine data on EOGBS or LOGBS incidence.^6^, ^7^, ^27^ In addition to our primary analysis, we used a Bayesian evidence synthesis model that combined direct incidence data with studies on maternal GBS colonisation and EOGBS risk conditional on GBS colonisation; this alternative approach resulted in a slightly lower estimated incidence. More studies with enhanced case capture, similar to one study in the UK, ^45^ and that also assess maternal GBS colonisation status would allow for a direct comparison between these approaches to inform burden estimation. Underascertainment and under-reporting also apply to LOGBS; hence, we estimated the region-specific proportions of iGBS cases occurring after the first week of life and applied these to the estimated numbers of EOGBS cases to calculate the LOGBS incidence. An underlying assumption is that EOGBS and LOGBS are equally likely to be identified in incidence studies; if EOGBS cases are missed more often than LOGBS cases, it is possible that our analysis might have overestimated the number of LOGBS cases.

Our analyses have strengths and limitations. We were able to advance the previous GBS global burden estimates,^7^ particularly with new studies on NDI from either high-income countries or low-income and middle-income countries,^29^ which were funded as a result of the gaps shown in the first study.^15^ The Bayesian approach has many advantages compared with the previous estimation approach, including the propagation of uncertainty for several parameter estimates, although, as mentioned earlier, a few of these parameters were assumed to be fixed.

However, there are still some data gaps. The sample sizes of some of the studies, in particular the studies on NDI outcomes after GBS meningitis in low-income and middle-income countries, were small, which highlights the difficulty in performing these studies in some settings and the need to improve surveillance to identify those who recover from iGBS.^29^ Intrapartum antibiotic prophylaxis coverage data are also few, and our analysis is informed by a literature review with data for 92 countries. Currently, our estimates do not incorporate uncertainty for this variable; but a sensitivity analysis (appendix pp 6–7) indicates how the overestimation or underestimation of intrapartum antibiotic prophylaxis coverage might have influenced estimation. Studies that quantify intrapartum antibiotic prophylaxis policy coverage in different countries would improve our estimates and help to better capture between-country variability. There were few data on maternal iGBS and related maternal mortality. We also did not quantify the global effect of iGBS on educational needs for children with GBS-associated NDI, a problem that was identified in a large cohort study in the Netherlands,^15^ because data from different settings are not available. Another limitation in our estimates is the few data on fatality rates during iGBS for children who have limited access to care and antibiotics. Although our assumptions were similar to those made in previous analyses, we also presented results where all children, including those with limited access to care, were assumed to have similar fatality rates during iGBS as those reported in observational studies. Furthermore, for parameters estimated at the global level (ie, assumed to be the same for all countries), it is probable that the uncertainty was underestimated and point estimates might have been biased when applied to specific settings. For example, more data on NDI after GBS meningitis were available for high-income countries, which implies, in addition to the potential bias (eg, an underestimation of NDI risk after GBS meningitis when this global risk is applied to low-income settings), that uncertainty in settings with fewer studies would be higher; data from low-income and middle-income countries are necessary to allow robust region-specific estimations. As is common in many burden estimation exercises, the data used to inform most outcomes were typically before the year of estimation (2020) and might have been influenced by the COVID-19 pandemic.

In summary, our estimates show that pregnancy-related GBS results in a considerable disease burden worldwide, with the highest absolute burden in sub-Saharan Africa and Asia. Effective interventions could reduce the high incidence of iGBS, and prevent the devastating long-term consequences on neurodevelopment, but also need to be implementable at high coverage in the wide range of settings where GBS infection is a public health problem; it is likely that maternal GBS vaccines could be more scalable than intrapartum antibiotic prophylaxis in the lowest resource settings. Interventions that have a preventive effect earlier in a pregnancy than intrapartum antibiotic prophylaxis, which is given around the time of birth, would have the added value of reducing stillbirths, maternal infections, and potentially GBS-associated preterm births. Our results can be used to refine cost-effectiveness analyses regarding maternal GBS vaccines under clinical development, through the inclusion of NDI and other long-term consequences of infection, in addition to mortality. Our data and analyses on NDI underline the necessity of follow-up and providing care to those who recover from iGBS. However, there are still substantial data gaps. We hope that before another round of estimates is undertaken, well designed studies could address the top epidemiological data gaps regarding stillbirths, maternal iGBS, and preterm risk.

## Data sharing

Datasets with published data used in the meta-analyses are available upon request (either Bronner Gonçalves or Proma Paul can be contacted; the emails are bronner.goncalves@lshtm.ac.uk and proma.paul@lshtm.ac.uk) or directly from the appendices of the systematic literature reviews cited in this paper. Unpublished data, including new datasets on NDI or stillbirths, from the CHAMPS network require communication with the investigators leading these specific studies.

## Declaration of interests

The Department of Clinical Epidemiology of Aarhus University receives funding from private and public institutions in the form of institutional research grants to (and administered by) Aarhus University; none of these grants has any relation to the present study. SAM declares funding from Astrazeneca, the Bill & Melinda Gates Foundation, GlaxoSmithKline, Minervax, Novavax, Pfizer, and the South Africa Medical Research Council; in particular, SAM delares funding to his institution from Pfizer for epidemiology studies on group B streptococcus (GBS) and a clinical trial on the GBS vaccine, and from the Bill & Melinda Gates Foundation on GBS epidemiology. FS declares employment by the UK National Screening Committee, which developed the policy recommendation for maternal GBS screening. CT declares a consulting fee from WHO for drafting a report on the Full Value of Vaccine Assessment for GBS vaccines, which is related to the current manuscript. RL declares participation on an advisory board for Janssen and Pfizer; payment for lectures from Reckitt; and grants to Fundación INFANT from the Bill & Melinda Gates Foundation and PATH. All other authors declare no competing interests.
