# Correction to *Lancet Glob Health* 2022; 10: e807–19

**DOI:** 10.1016/S2214-109X(22)00235-2

**Published:** 2022-05-12

*Gonçalves BP, Procter SR, Paul P, et al. Group B streptococcus infection during pregnancy and infancy: estimates of regional and global burden.* Lancet Glob Health *2022;* 10: *e807–19*—An administrative error led to incomplete listing of the collaborative group on PubMed; this has been corrected as of May 12, 2022.

