# Supplementary material for: Group B streptococcus infection during pregnancy and infancy: estimates of regional and global burden
Source: Lancet Glob Health. 2022 Apr 28;10(6):e807–19. doi: 10.1016/S2214-109X(22)00093-6 (PMC9090904; doi:10.1016/S2214-109X(22)00093-6)
Supplement: Supplementary appendix [file mmc1.pdf]

THE LANCET
Global Health

Supplementary appendix

This appendix formed part of the original submission and has been peer reviewed. We post it as supplied by the authors.

Supplement to: Gonçalves BP, Procter SR, Paul P, et al. Group B streptococcus infection during pregnancy and infancy: estimates of regional and global burden. *Lancet Glob Health* 2022; published online April 28. https://doi.org/10.1016/S2214-109X(22)00093-6.

Title: Group B Streptococcus infection during pregnancy and infancy: estimates of regional and global burden

Contents

GATHER statement checklist 2

Methods Supplement - Details per parameter 4

Maternal GBS colonisation 4

Early-onset invasive GBS disease 6

Late-onset invasive GBS disease 8

Mortality during iGBS (CFR) 10

Neurodevelopmental impairment after iGBS 13

Stillbirths attributed to GBS 17

iGBS Maternal disease 20

Preterm birth associated with maternal GBS colonisation 21

Computational methods 23

Results Supplement 24

Figures 24

Tables 35

References 45

GATHER statement checklist

This table was downloaded from http://gather-statement.org/ and adapted.

**Objectives and funding**		
**1**	Define the indicator(s), populations (including age, sex, and geographic entities), and time period(s) for which estimates were made.	Introduction, Methods
**2**	List the funding sources for the work.	Abstract
**Data Inputs**		
For all data inputs from multiple sources that are synthesized as part of the study:		
**3**	Describe how the data were identified and how the data were accessed.	Methods, Supplementary Methods
**4**	Specify the inclusion and exclusion criteria. Identify all ad-hoc exclusions.	Primarily based on literature reviews (described in Supplementary Methods).
**5**	Provide information on all included data sources and their main characteristics. For each data source used, report reference information or contact name/institution, population represented, data collection method, year(s) of data collection, sex and age range, diagnostic criteria or measurement method, and sample size, as relevant.	Table 1, Methods and Supplementary Methods. Detailed information on the different studies can be obtained from the supplementary appendices of the different literature reviews referenced in the paper and below.
**6**	Identify and describe any categories of input data that have potentially important biases (e.g., based on characteristics listed in item 5).	Limitations of data and model assumptions are described in Table S9 and in the Discussion section.
For data inputs that contribute to the analysis but were not synthesized as part of the study:		
**7**	Describe and give sources for any other data inputs.	This information is presented in Table 1 and in the Methods and Supplementary Methods.
For all data inputs:		
**8**	Provide all data inputs in a file format from which data can be efficiently extracted (e.g., a spreadsheet rather than a PDF), including all relevant meta-data listed in item 5. For any data inputs that cannot be shared because of ethical or legal reasons, such as third-party ownership, provide a contact name or the name of the institution that retains the right to the data.	Published data used in meta-analyses have been uploaded in a data repository. Some of the datasets are also available in data repositories from authors who performed the literature reviews or in the supplementary appendices of the reviews. Access to unpublished data requires direct communication with leading investigators of specific studies (e.g. for new data on NDI after iGBS).
**Data analysis**		
**9**	Provide a conceptual overview of the data analysis method. A diagram may be helpful.	Methods and Supplementary Methods describe the statistical approach in detail. Figure 1 shows the different outcomes being modelled.

**10**	Provide a detailed description of all steps of the analysis, including mathematical formulae. This description should cover, as relevant, data cleaning, data pre-processing, data adjustments and weighting of data sources, and mathematical or statistical model(s).	This information is in the Methods section and the Supplementary Methods.
**11**	Describe how candidate models were evaluated and how the final model(s) were selected.	In the Supplementary Methods, we describe model checks, sensitivity analyses, and secondary analyses for some of the outcomes; see also in [1].
**12**	Provide the results of an evaluation of model performance, if done, as well as the results of any relevant sensitivity analysis.	Supplementary Methods (including figures) and Table S8
**13**	Describe methods for calculating uncertainty of the estimates. State which sources of uncertainty were, and were not, accounted for in the uncertainty analysis.	Methods, Results and Supplementary Methods
**14**	State how analytic or statistical source code used to generate estimates can be accessed.	Statement in the Supplementary Methods
**Results and Discussion**		
**15**	Provide published estimates in a file format from which data can be efficiently extracted.	Summary tables (Tables in the main manuscript) are available in a data repository
**16**	Report a quantitative measure of the uncertainty of the estimates (e.g. uncertainty intervals).	Results section
**17**	Interpret results in light of existing evidence. If updating a previous set of estimates, describe the reasons for changes in estimates.	Discussion section
**18**	Discuss limitations of the estimates. Include a discussion of any modelling assumptions or data limitations that affect interpretation of the estimates.	Table S9, Methods, Results, Discussion sections, Supplementary Methods

Methods Supplement - Details per parameter

This section is subdivided in subsections: Maternal GBS colonisation; Early-onset invasive GBS disease; Late-onset invasive GBS disease; Mortality during invasive GBS disease; Neurodevelopmental impairment after invasive GBS disease; Stillbirths attributed to GBS; Maternal disease; Preterm birth associated with maternal GBS colonisation. In each subsection, we describe the data used, present the model used in our analysis, and include discussions on prior assumptions when relevant.

Maternal GBS colonisation

We developed a Bayesian hierarchical model to estimate country-level maternal GBS colonisation prevalence and its association with relevant country-level variables. A total of 325 data points, from 82 countries, directly informed this estimation; these data were reviewed in [2], where GBS colonisation was defined based on culture results. The model below shows how data from prevalence studies informed national estimates:

Country level

$\mu_m \sim Normal\ (\mu_{g-c} + \beta_c\, X_m^c, \sigma_c^2\ )$

Study level

$logit\ (p_{im}^{col}) \sim Normal\ (\ \mu_m + \beta_s\, X_{im}^S\ , \sigma_s^2\ )$

$y_{im}^{col} \sim Binomial\ (p_{im}^{col}, n_{im}^{col}\ )$

Priors

$\beta_c \sim Normal\ (0, 1)$

$\beta_s \sim Normal\ (0, 1)$

$\mu_{g-c} \sim Normal\ (-1, 1)$

$\sigma_c \sim Uniform\ (0, 5)$

$\sigma_s \sim Uniform\ (0, 5)$

The logit-prevalence in a country m, μ_m, was assumed to depend on the global intercept, μ_{g-c}, and country-level variables, X_m^c, that were standardised before analysis; β_c represents a vector of regression coefficients. The following country-level variables were used based on previous analyses[1,3]: prevalence of female obesity, gross national income per capita, HIV prevalence, neonatal mortality, antibiotics coverage during lower respiratory tract infections in children (as a proxy for antibiotics availability and usage in each country), and maternal education. Missing values in country-level variables were input using mean values in each WHO region. The prevalence p_{im}^{col} in studies was modelled as a function of two binary study-level variables (anatomical sampling site and microbiological method), where X_{im}^S is the vector of covariate values for study i in country m; the prevalence is also dependent on the country where the study was performed through the intercept μ_m. Study-level covariates were coded so higher sensitivity procedures had value 0, and lower sensitivity methods, value 1. We assumed common within-country between-study variance, σ_s^2. For each study i performed in country m, the number of colonized pregnant women, y_{im}^{col}, was assumed to follow a binomial distribution. The parameters of each of these distributions were n_{im}^{col}, the study sample size, and p_{im}^{col}. Priors used for this analysis were: for regression coefficients, the β's, we used weakly informative normal priors, *Normal ~ (0, 1)*; for μ_{g-c}, the prior *Normal ~ (-1, 1)* was used; *Uniform ~ (0, 5)* was used for scale parameters, σ's. In particular, we used priors consistent with our knowledge that generally less than half of the maternal population is colonised by GBS. In **Table S2**, we present posterior estimates of model coefficients. Posterior distributions of the coefficients were used in the estimation of maternal GBS colonization prevalence for countries without data; this also incorporates the unexplained between-country variation represented by the scale parameter σ_c. Region-specific numbers of colonised mothers, as reported in **Figure 2**, were calculated by multiplying country-specific numbers of births and the inverse-logit of the corresponding parameter μ_m for each posterior sample.

Note that predictive checks for the model on country-level GBS colonisation prevalence and for the model described in the following subsection, on early-onset invasive GBS disease risk, are presented in [1].

Early-onset invasive GBS disease

Thirty studies with at least 200 GBS-colonised pregnant women were identified in a recent review[4]; two studies with only term babies were not included in this analysis, as it is possible risks in these studies do not reflect risk in the general population[5]. Early-onset invasive GBS disease (EOGBS) was defined based on blood or cerebrospinal fluid culture. The model is described below:

$$\alpha_i \sim Normal(\alpha_{g-eo}, \sigma_{eo}^2)$$

$$logit\ (r_i) = \alpha_i + \beta_{IAP}\ X_i^{IAP}$$

$$y_i^{eo} \sim Binomial\ (r_i, n_i^{eo}\ )$$

Priors

$$\alpha_{g-eo} \sim Normal\ (-4, 1)$$

$$\beta_{IAP} \sim Normal\ (0, 1)$$

$$\sigma_{eo} \sim Uniform\ (0, 5)$$

Here, y_i^{eo} represents the number of early-onset GBS cases in study *i*. This number follows a binomial distribution with parameters n_i^{eo}, sample size of study *i* (i.e. number of GBS colonised mothers), and r_i, risk in the study *i*. α_i are study-specific intercepts, normally distributed with location parameter α_{g-eo} and scale σ_{eo}. β_{IAP} is the coefficient of the association between study-level intrapartum antibiotic use and risk of EOGBS. Priors were: for α_{g-eo}, *Normal ~ (-4, 1)*, which corresponds to the expected low risk of iGBS disease[6]; *Uniform ~ (0, 5)* and *Normal ~ (0, 1)* were used for σ_{eo} and β_{IAP}. Prior predictive distribution for this model is presented in [1].

To estimate country-level numbers of EOGBS cases, in addition to country-specific live births in GBS-colonised mothers, which were calculated based on total number of births, stillbirth risk and estimated GBS colonisation prevalence, we used $inverse\_logit\ (\alpha_{g-eo} + \beta_{IAP}\ X_m^{IAP})$, where X_m^{IAP} is country-specific intrapartum antibiotic prophylaxis (IAP) coverage estimated by Le Doare and colleagues[7], and

which was incorporated in our analysis as fixed value rather than uncertain quantity. Of note, for countries for which Le Doare and colleagues did not estimate IAP coverage, we assumed zero coverage of IAP in developing countries (N = 87), as this was the coverage in 56% of developing countries with estimated coverage, and assumed 80% coverage in developed countries (N = 16). By assuming fixed values, our results reflect uncertainty in other parameters but not in this parameter. As a sensitivity analysis, if IAP coverages in countries with above-zero (assumed or estimated) coverage were for example (i) 5% higher or (ii) 5% lower than our current assumptions, estimated numbers of EOGBS cases would be 224,00 (110,600 – 451,500) and 240,000 (111,900 – 477,500), respectively.

We also performed a secondary analysis that combined information on maternal GBS colonisation prevalence, risk of EOGBS in babies born to mothers with GBS colonisation and data from studies with direct incidence estimates in all births. These different study types were linked using a parameter that corresponds to the probability of reporting incident cases, assumed common for all incidence studies, and through functions of parameters estimated in this and the previous section, including the β_{IAP} parameter. Ten incidence studies considered to be less subject to bias and described in [8] were included. The equation used is shown below and described in detail in [1] (**Table S3**):

$$u_i = p'_{im} \times \; inverse\_logit\left(\alpha'_i + \; \beta_{IAP} X_{im}^{IAP}\right) \times \; \gamma$$

where u_i represents the underlying risk of confirmed EOGBS in all births in incidence study *i*; γ is the aforementioned parameter that corresponds to reporting probability; and α'_i and p'_{im} are, respectively, the estimated study-specific intercept for the risk of EOGBS given colonisation in study *i*, and estimated study-specific prevalence of maternal GBS colonisation based on the model in the previous section. X_{im}^{IAP} represents IAP coverage in country *m*, assumed to correspond to coverage in the incidence study *i* population.

Whilst this framework allowed inclusion of additional data in the estimation, calculation of country-specific numbers of EOGBS cases was as outlined above. In the different analyses described in this subsection, we do not discriminate between microbiological- and risk factor-based IAP approaches; this might have led to underestimation of EOGBS risk in countries with primarily risk factor-based IAP policy.

Late-onset invasive GBS disease

It has been argued that incidence studies, i.e. studies that directly estimate incidence of iGBS in all births, only capture a fraction of all incident cases. Evidence comes from studies that estimated under-reporting (e.g. an early study by Heath and colleagues in the United Kingdom[9]), from a review on GBS incidence studies performed in low- and middle-income countries[10] and from individual studies that discuss under-ascertainment and/or under-reporting[11]. Under-estimation is thought to occur, probably due to different reasons, for both EOGBS and late-onset invasive GBS disease (LOGBS) incidence. Although it is possible that the degree of under-estimation varies depending on timing of disease onset, here we assumed that incidence under-estimation was of similar degree for both EOGBS and LOGBS. Whilst it was possible to indirectly estimate number of EOGBS cases by combining maternal GBS colonisation prevalence estimates and estimates on the risk of EOGBS given maternal GBS colonisation, the same approach cannot be used for LOGBS. For this reason, for incidence studies with appropriate follow-up duration and that assessed the incidence of both EOGBS and LOGBS cases (N = 20, reviewed in [8]), we estimated relative frequencies of these presentations and applied them to our estimates of country-specific EOGBS incidence. The following model was used:

$$\mu_j^{lo} \sim Normal\ (\mu_{g-lo}, \sigma_{g-lo}^2)$$
$$logit\left(p_{ij}^{lo}\right) \sim Normal\ (\mu_j^{lo}, \sigma_{s-lo}^2)$$
$$y_{ij}^{lo} \sim Binomial\ (p_{ij}^{lo}, n_{ij}^{lo})$$

Priors

$$\mu_{g-lo} \sim Normal\ (0, 1)$$

$$\sigma_{g-lo} \sim Normal\ (0, 1)$$

$$\sigma_{s-lo} \sim Normal\ (0, 1)$$

The total number of iGBS cases in each study is n_{ij}^{lo}. We assumed the number of LOGBS cases in the study *i* performed in region *j* (y_{ij}^{lo}) follows a binomial distribution, with study-specific logit-proportion ($logit\left(p_{ij}^{lo}\right)$) being normally distributed with location parameter corresponding to the region-specific

proportion at the logit scale, μ_j^{lo}. Posterior estimates are shown in **Figure S3**. We assumed the following priors: $\mu_{g-lo} \sim Normal\ (0,\ 1)$ and $\sigma_{g-lo} \sim Normal\ (0,\ 1)$. Note that here and throughout this analysis, whenever a Normal distribution was used as prior for a scale parameter, the parameter was constrained to be positive. Prior and mixed predictive checks were performed. The mixed predictive distribution, generated by using posterior samples of μ_j^{lo} and σ_{s-lo} to sample $logit\ (p_{ij}^{lo})$ for new studies in each region, is shown below:

Figure legend. The top panel shows median (black circle) and percentile intervals (2.5 – 97.5, 25 – 75, and 40 – 60%), represented by different tones of red, of the mixed predictive distribution for each study (x-axis); black stars correspond to observed values. For each replicated dataset generated from the mixed predictive distribution, we calculated the width of the range of proportions in replicated studies; this is plotted in the bottom panel, with the range width observed in studies included in the analysis (vertical dashed line).

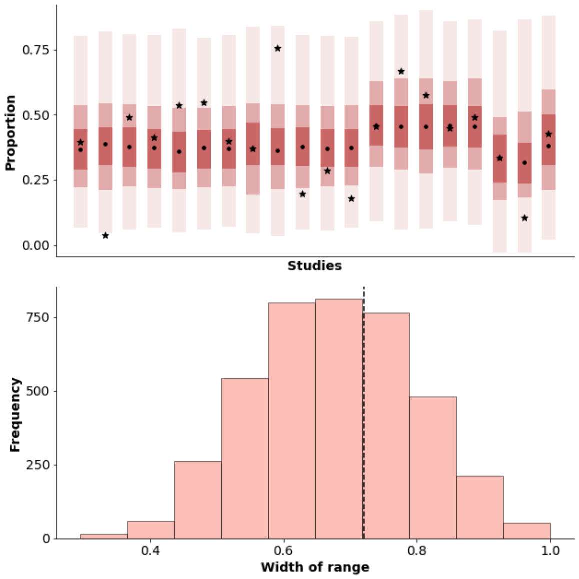

Estimated region-specific proportions of iGBS cases presenting as LOGBS, $inverse\_logit(\mu_j^{lo})$, were used to calculate multiplication factors ($\frac{inverse\_logit(\mu_j^{lo})}{(1-inverse\_logit(\mu_j^{lo}))}$) that were applied to country-specific numbers of EOGBS cases, as estimated in the previous section.

Mortality during iGBS (CFR)

Data from 47 and 29 studies were used to estimate case fatality rates (CFR) of EOGBS and LOGBS cases, respectively. The number of EOGBS cases in these studies ranged from 1 to 517 and of LOGBS, from 3 to 373. In addition to analyses including all these studies, reviewed in [8], we also performed a sensitivity analysis that only included studies with more than 10 cases and with appropriate follow-up (i.e., 0 – 6 or 0 – 7 days for EOGBS data and 7 – 89 or 7 – 90 days for LOGBS data). These estimates were not dissimilar to results presented in **Table S5**, except: EOGBS CFR in Latin America and Caribbean region was lower than in countries in the developed group (posterior medians ~2 versus ~6%). We estimated region-specific CFR using the model below:

$$\mu_j^{cfr} \sim Normal\ (\mu_{g-cfr}, \sigma_{g-cfr}^2)$$
$$logit\ (p_{ij}^{cfr}) \sim Normal\ (\mu_j^{cfr}, \sigma_{s-cfr}^2)$$
$$y_{ij}^{cfr} \sim Binomial\ (p_{ij}^{cfr}, n_{ij}^{cfr})$$

Priors

$$\mu_{g-cfr} \sim Normal\ (-1, 1)$$
$$\sigma_{g-cfr} \sim Normal\ (0, 1)$$
$$\sigma_{s-cfr} \sim Normal\ (0, 1)$$

Whilst model structure was similar for EOGBS and LOGBS, two separate models were used since EOGBS and LOGBS have different compositions of clinical syndromes (sepsis and meningitis). The following description is valid for both models: μ_j^{crf} represents region-specific logit-CFR, and p_{ij}^{cfr}, study-specific CFR; σ_{g-cfr} and σ_{s-cfr} are scale parameters that represent between-region and within-region between-study variations. Posterior estimates are shown in **Table S5** and **Figure S4**.

For both EOGBS and LOGBS CFR models, we assumed the following priors: $\mu_{g-cfr} \sim Normal\ (-1, 1)$ and $\sigma_{g-cfr} \sim Normal\ (0, 1)$. Estimates using other prior assumptions are presented in **Table S8**.

Below we show the prior predictive distribution for the EOGBS CFR model, which suggests most datasets compatible with our prior assumption would have higher-than-observed CFR. However, as shown in **Table S8**, similar results are obtained with other prior assumptions.

Figure legend. This figure shows the prior predictive distribution for studies with sample sizes that correspond to sample sizes included in the estimation; different shades in blue represent the following pairs of percentiles: 2.5 – 97.5, 25 – 75, and 40 – 60. Stars represent observed CFR in different studies.

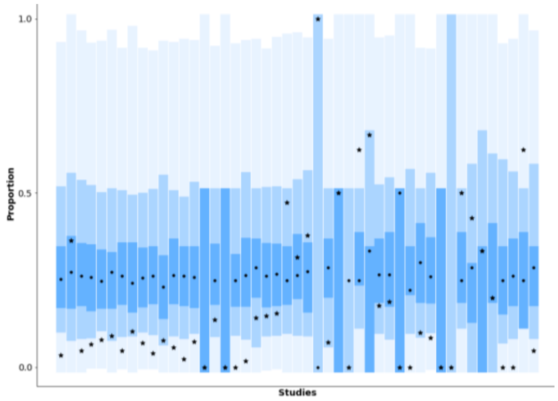

Below the mixed predictive distribution for EOGBS CFR is shown; here CFRs in new studies in each region were sampled using posterior samples of μ_j^{cfr} and σ_{s-cfr} .

Figure legend. The figure shows median (black circle) and percentile intervals (2.5 – 97.5, 25 – 75, and 40 – 60%), represented by different tones of red, of the mixed predictive distribution for each study (x-axis); black stars correspond to observed values.

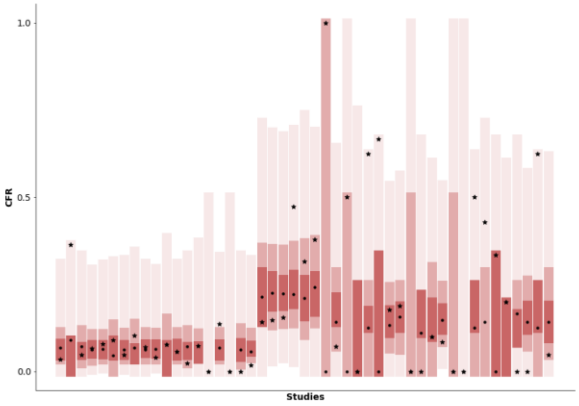

Of note, we also fit a model with region-specific scale parameters for EOGBS CFR and obtained similar results.

Region-specific CFR ($inverse\_logit(\mu_j^{cfr})$) were applied to estimated numbers of cases (either EOGBS or LOGBS). To estimate the number of deaths in children who developed iGBS in 2020, we also used information on skilled birth attendance coverage. We assumed that children without skilled birth attendance would suffer high mortality, a fixed value of 90%, if they developed EOGBS. This is similar to the average mortality assumed by Seale et al[3], although here we did not introduce uncertainty in this parameter; as stated in the manuscript, uncertainty intervals thus do not reflect our lack of data on this risk. For children who had access to skilled birth attendance and developed EOGBS and for all children who developed LOGBS, we applied the CRFs estimated above. For five countries, skilled birth attendance data were missing and we used the regional median.

Neurodevelopmental impairment after iGBS

Since most studies assessing long-term risk of neurodevelopmental impairment after iGBS did not include a comparator group, to use data from studies reviewed in [12], in addition to data from a large cohort study in Denmark[13], we conducted a meta-analysis of the risk of impairment in children with history of iGBS, rather than of the association between iGBS and neurodevelopmental impairment (NDI). Unpublished data (Proma Paul, personal communication) collected in five low- and middle-income countries (Argentina, India, Kenya, Mozambique, South Africa) on NDI risk after iGBS were also used in this estimation; these data (henceforth, LMIC-NDI data) were collected in a recent multi-centre study that used multiple direct assessment tools[14] and a multi-domain definition of NDI[15]. As mentioned in the manuscript, data from the Argentinian study were not included in this analysis as the low proportion of iGBS survivors identified who were assessed might have been linked to selection bias. In the study in South Africa, a similarly low proportion of eligible iGBS survivors had NDI assessment; however, the clinical characteristics of these children did not differ significantly from those of a larger cohort of iGBS survivors of which they were part.

As recent data suggest[13], risk of NDI varies by GBS syndrome; for this reason, we first estimated the distribution of iGBS survivors by clinical presentation (sepsis and meningitis). We included 24 and 14 studies with data on the proportions of EOGBS and LOGBS cases, respectively, diagnosed as meningitis[8]. This was modelled as:

$$logit\ (p_i^{men}) \sim Normal\ (\ \mu_{g-men}\ , \sigma_{g-men}^2\ )$$
$$y_i^{men} \sim Binomial\ (p_i^{men}, n_i^{men}\ )$$

Priors

$$\mu_{g-men} \sim Normal\ (-1, 1)$$

$$\sigma_{g-men} \sim Normal\ (0, 1)$$

Proportions of EOGBS and LOGBS cases that were meningitis cases were modelled separately. For each of these models, y_i^{men} represents the number of meningitis cases in study *i*; and n_i^{men} is the total number of EOGBS (or LOGBS) cases in the study. A separate hyperparameter μ_{g-men} was estimated for EOGBS

and LOGBS. Overall proportions, $inverse\_logit(\mu_{g-men})$, were then applied to numbers of children who survived the acute episode (see previous subsection). In this analysis we assumed the following priors for both EOGBS and LOGBS: $\mu_{g-men} \sim Normal\ (-1\ ,1)$ and $\sigma_{g-men} \sim Normal\ (0\ ,1)$. Sensitivity analyses with different prior assumptions were performed; similar results were obtained (results not shown). Our models suggest that 12.9% (9.5 – 17.2) and 40.6 % (30.3 – 52.0) of EOGBS and LOGBS cases, respectively, present as meningitis. Note that for this estimation only numbers of GBS sepsis and GBS meningitis cases were used, including in studies that reported other clinical manifestations.

After estimating numbers of iGBS survivors by syndrome, we applied syndrome-specific risks of NDI to quantify the total number of moderate and severe NDI cases. Fifteen studies reviewed by Kohli-Lynch et al[12], data from the Danish cohort described in [1] and the unpublished LMIC-NDI data informed estimations for children who survived GBS meningitis; since the number of participants with history of GBS meningitis was limited in the LMIC-NDI data (2 of the 4 sites had fewer than 10 participants with GBS meningitis), we estimated a global risk of NDI after GBS meningitis, rather than region-specific risks. To quantify the risk of NDI after GBS sepsis, data were available from 5 studies performed in high-income countries, four described in the Supplementary Appendix of a recent systematic review[12] and the Danish cohort study mentioned above; a meta-analysis of these studies was performed in estimating NDI risk after GBS sepsis in high-income countries. The LMIC-NDI data, specifically generated to address the data gap on NDI risk related to GBS sepsis in low- and middle-income countries, were used to model risk of NDI in these countries (range 22 – 31 participants with history of GBS sepsis). In addition to modelling risks of moderate and severe impairment, for each syndrome and also for each country group (high-income and low- and middle-income groups) in the GBS sepsis-specific estimation, we also model risk of any severity impairment, which includes milder forms of impairment. However, results for the latter estimation are only presented below, not in the main text, because, given the variation in study design, case ascertainment and methods used to diagnose NDI, we believe risk of moderate and severe impairment is the most appropriate outcome to be reported as it is more likely to be consistent across studies and settings.

Figures S5 and **S6** show posterior estimates of different risks. For the analysis on impairment after GBS meningitis, the following priors were used: $\mu_{g-syn}^{NDI} \sim Normal\ (-1\ ,1)$, which is consistent with the NDI risk after neonatal meningitis,[16] and $\sigma_{g-syn} \sim Normal\ (0\ ,1)$. For NDI after GBS sepsis, we used the

following prior assumptions: $\mu_{g-syn}^{NDI} \sim Normal\,(-2\,,4)$ and $\sigma_{g-syn} \sim Normal\,(0\,,1)$, which corresponds to the expectation that NDI risk after GBS sepsis is lower than the risk after GBS meningitis.

Below we show the prior predictive distribution for one of the models. As can be seen in **Table S8**, prior assumptions for the parameter σ_{g-syn} in the analysis on GBS sepsis in low- and middle-income countries, that involves a small number of studies, have an effect on the uncertainty of estimates.

Figure legend. This figure shows simulations from the prior predictive distribution for studies with sample sizes that correspond to sample sizes included in the estimation; the different shades in blue represent the following pairs of percentiles: 2.5 – 97.5, 25 – 75, and 40 – 60. Stars represent observed proportions of children with moderate and severe NDI in different studies.

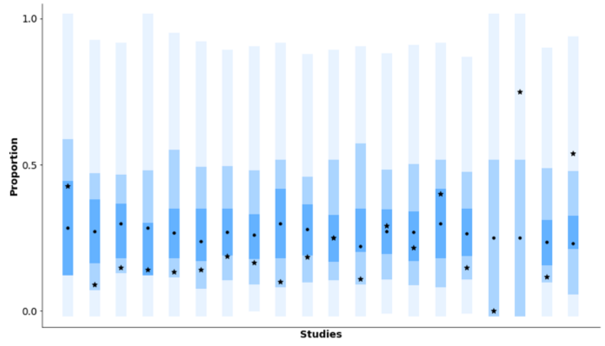

Figure legend. The figure shows median (black circle) and percentile intervals (2.5 – 97.5, 25 – 75, and 40 – 60%), represented by different tones of red, of the mixed predictive distribution for each study (x-axis); black stars correspond to observed values in GBS meningitis data.

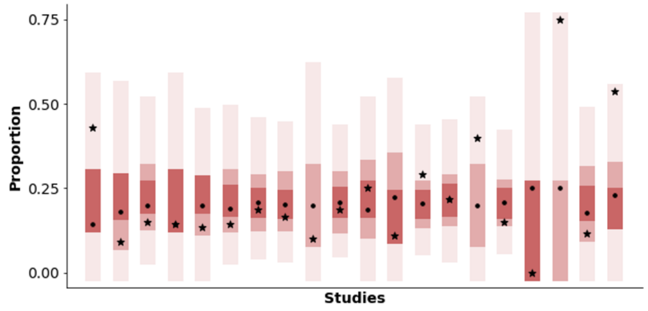

As mentioned above, we also estimated risk of NDI of any severity after iGBS: 35.2% (29.0 – 42.9) of those with GBS meningitis, 7.3% (2.5 – 16.9) with GBS sepsis in high-income countries and 36.8% (13.4 – 63.3)

with GBS sepsis in low- and middle-income countries are predicted to develop NDI (any severity). Applying these risks to numbers of survivors, we estimated 107,000 NDI cases (42,600 – 253,900).

In a secondary analysis that combines all the data on NDI risk after GBS sepsis, i.e. combines studies from low- and middle-income countries and high-income countries, risk of moderate and severe NDI was 4.8% (1.7 – 11.1) and the estimated number of moderate and severe NDI cases was 27,700 (12,100 – 64,600).

These different models allow the estimation of the total number of NDI cases in children who developed iGBS in 2020. However, it is likely that some of these children would have impairment even if they had not developed iGBS – in other words, not all NDI cases in children who have a history of iGBS are attributable to GBS. Here we also estimate the excess number of impairment cases in iGBS survivors by using the risk of moderate and severe NDI in children with no history of iGBS as estimated in the recent Danish cohort study; for that, instead of using the estimated risk post-GBS ($inverse\_logit(\mu_{g-syn}^{NDI})$), we applied the baseline risk to syndrome-specific numbers of survivors, and subtracted NDI cases estimated using the baseline risk from the total estimated using meta-analyses results. Since the baseline risk is likely variable or context-specific, results should be interpreted as only providing a crude estimation of excess number of NDI cases associated with iGBS. Of note, posterior samples of the risk of moderate and severe NDI after GBS sepsis overlapped with baseline risk, implying that for some posterior samples intermediate calculations only based on GBS sepsis survivors would lead to negative values. As a sensitivity analysis, we calculated excess number of impairment cases using as baseline risk values 50% lower (1.05%) and 50% higher (3.15%) than the value assumed in the primary analysis: 33,600 (12,700 – 90,100) and 27,100 (8,700 – 77,800), respectively.

Stillbirths attributed to GBS

To our knowledge, data are not available on the association between stillbirth risk and maternal GBS colonization; for this reason, our approach was to model the proportion of all stillbirths with GBS infection. Six studies performed after 2000 on the proportion of stillbirths with evidence of iGBS, and reviewed in [17], were included in this analysis; of note, one of the six studies also included data collected before 2000s. The underlying assumption is that detection of GBS in stillbirth tissues, as opposed to just skin, implies causality. The number of participants in these studies ranged from 18 to 5,175. Furthermore, data from the Child Health and Mortality Prevention Surveillance (CHAMPS) network [18] were also used. These data were requested in March 2021 and included information on 509 stillbirths investigated in six African countries and Bangladesh. In the CHAMPS network, the definition of GBS-related stillbirth required assessment by a panel of local investigators on whether the detected GBS, either by culture, molecular methods or immunohistochemistry, was deemed a significant factor in the chain of events leading to death. We used a hierarchical model to estimate proportions of stillbirths related to GBS in different regions (see below). Given independent estimates of country-specific stillbirth risk[19], we used these proportions to calculate stillbirth numbers caused by GBS in each country.

$$\mu_j^{stil} \sim Normal\ (\mu_{g-stil}, \sigma_{g-stil}^2)$$

$$logit\left(p_{ij}^{stil}\right) \sim Normal\ (\ \mu_j^{stil}\ , \sigma_{s-stil}^2\ )$$

$$y_{ij}^{stil} \sim Binomial\ (p_{ij}^{stil}, n_{ij}^{stil}\ )$$

Priors

$$\mu_{g-stil} \sim Normal\ (-3, 4)$$

$$\sigma_{g-stil} \sim Normal\ (0, 1)$$

$$\sigma_{s-stil} \sim Normal\ (0, 1)$$

where μ_j^{stil} represents the logit-proportion of stillbirths with GBS in region *j*, and p_{ij}^{stil}, the proportion in study *i* of region *j*. σ_{s-stil}^2 corresponds to within-region between-study variance. Using this model, we estimated proportions of stillbirths related to GBS in regions without studies, Oceania and Latin America, from the distribution defined by the location and scale hyperparameters μ_{g-stil} and σ_{g-stil}. In addition

to using Normal distributions, we also fit this model using Student-t distribution with 4 degrees of freedom; since results were similar, these are not presented.

Below we present the prior predictive distribution using the following assumptions: μ_{g-stil} ~ *Normal (-3, 4)*, which corresponds to having most of the prior probability distribution between 0.06 and ~25%, consistent with results of studies performed before 2000 and reviewed by Seale and colleagues; and σ_{g-stil} ~ *Normal* (0,1), which is based on the assumption that between region variation is limited.

Figure legend. In this figure, the top panel shows simulations from the prior predictive distribution for studies with sample sizes that correspond to sample sizes included in the estimation; the different shades in blue represent the following pairs of percentiles: 2.5 – 97.5, 25 – 75, and 40 – 60. The bottom panel shows the same information as a histogram; in this plot each line represents prior predictions for a different study.

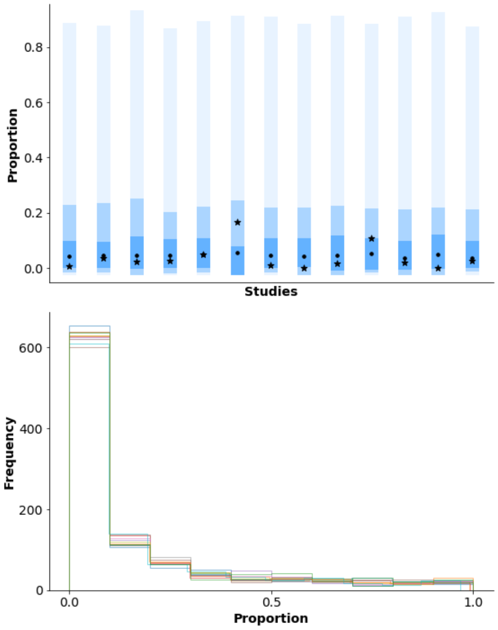

The posterior predictive distribution is presented below. Here mixed predictions were used, where μ_{j-new}^{stil} was sampled from $Normal\ (\mu_{g-stil}\ ,\sigma_{g-stil}^{2}\ )$ using posterior samples of μ_{g-stil} and σ_{g-stil}. $logit\ (p_{ij}^{stil})$ was sampled for each study using μ_{j-new}^{stil} and posterior samples of σ_{s-stil}.

Figure legend. The top panel shows the mixed predictive distribution for each study, with the median presented as a black circle and the observed value, a black star. The bottom panel shows the maximum proportion of GBS-related stillbirths in replicated datasets and the vertical dashed line corresponds to the observed maximum value.

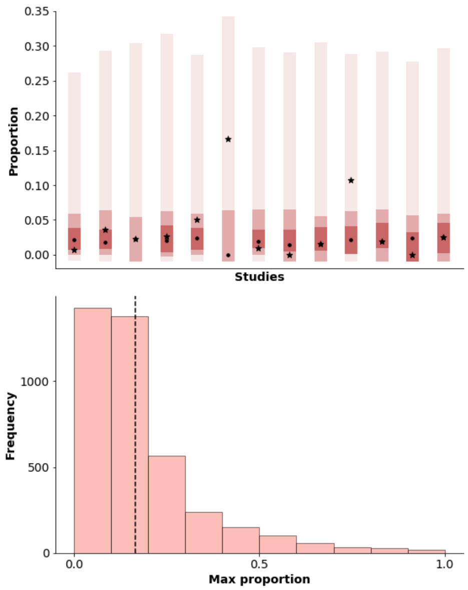

Posterior estimates are presented in **Figure S2** and **Table S7**. We also fit a model with region-specific between-study scale parameters; since results were similar, they are not presented.

In analyses described in the preceding subsections, country-specific numbers of stillbirths were subtracted from country-specific numbers of births; in particular, all stillbirths estimated to be linked to GBS were subtracted from numbers of GBS-colonised pregnant women.

iGBS Maternal disease

Four studies were identified in a recent review[20] that provided information on the risk of maternal GBS disease; three of these studies had as denominator deliveries, whilst, the other, pregnancies. In addition to these studies, we included a study published by Collin et al on the incidence of GBS-associated maternal morbidity in 2015 and 2016 in England[21]. The hierarchical model used was:

$$logit\ (p_i^{mat})\ \sim Normal\ (\mu_{g-mat}, \sigma^2_{g-mat})$$
$$y_i^{mat} \sim Binomial\ (p_i^{mat}, n_i^{mat})$$

Priors

$$\mu_{g-mat} \sim Normal\ (-8, 1)$$

$$\sigma_{g-mat} \sim Normal\ (0, 1)$$

The prior predictive distribution is shown below; and posterior estimates, in **Figure S8**. Priors were: $\mu_{g-mat} \sim Normal\ (-8, 1)$, which is consistent with fewer than 2.5 cases per 1,000 deliveries, and $\sigma_{g-mat} \sim Normal\ (0, 1)$. Alternative prior assumptions are shown in **Table S8**. Numbers of cases were estimated by applying the overall risk $inverse\_logit(\mu_{g-mat})$ to the number of births in each country.

Figure legend. This figure shows the prior predictive distribution as a histogram; in this plot each line represents prior predictions for a different study.

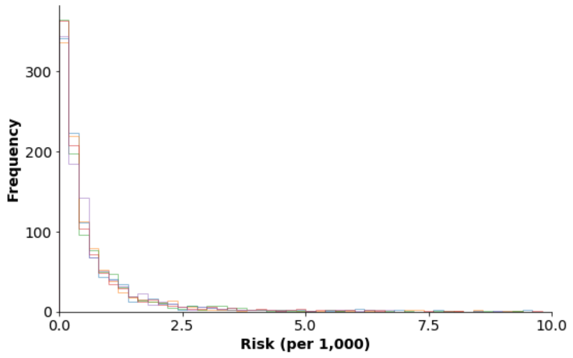

Preterm birth associated with maternal GBS colonisation

GBS might also indirectly cause morbidity and mortality by increasing the risk of prematurity. Here we used data reviewed by Bianchi-Jassir and colleagues[22] to quantify this association. As in the review, we excluded studies that used urine samples.

Firstly, we performed a Bayesian random-effects meta-analysis of case-control studies. The estimated odds ratio (posterior median and 95% interval) was 1.83 (1.04 – 3.07).

Meta-analysis of case-control studies

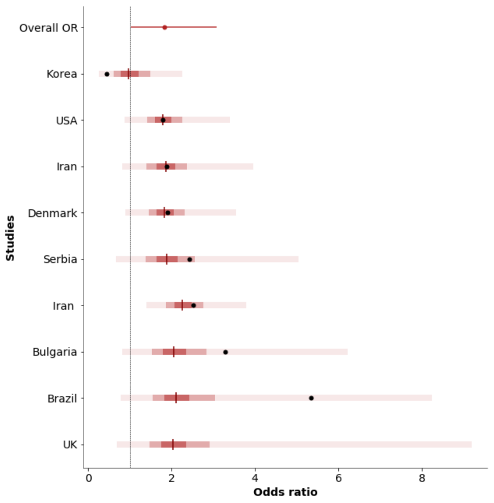

Posterior mean and standard deviation of the random effects location parameter in the case-control meta-analysis were used as priors in the analysis of the other study types. Data from 28 studies were used in the meta-analysis of cohort and cross-sectional studies (**Figure S7**):

$$\beta_g^{pret} \sim Normal\ (\ 0.59\ , 0.27^2\ \ )$$

$$\beta_i^{pret} \sim Normal\ (\ \beta_g^{pret}\ , \sigma_{g-pret}^2\ )$$

$$logit\ (p_{it}^{pret}) = \mu_i^{pret} + \beta_i^{pret} x_{it}$$

$$y_{it}^{pret} \sim Binomial\ (p_{it}^{pret}, n_{it}^{pret}\ )$$

β_i^{pret} represents study-specific coefficients of the association between maternal GBS colonisation and prematurity; μ_i^{pret} are study-specific intercepts; p_{it}^{pret} are study- and group (indexed by *t*, the colonisation status)-specific probabilities of prematurity; x_{it} is 0 for babies born to mothers who are not GBS colonised and 1, for babies of mothers who are.

We used the odds ratio, exponential (β_g^{pret}), as the measure of association. National estimates of preterm risk [23] were used, together with the odds ratio and country-specific maternal GBS colonisation prevalence, to calculate the excess number of preterm births associated with GBS colonisation. Two approaches were used: in the first, population attributable fraction formula was used; in the second approach, we solved the system of equations below for each posterior sample:

$$OR = \left(\frac{p_j^{exp}}{1 - p_j^{exp}}\right)\left(\frac{1 - p_j^{unexp}}{p_j^{unexp}}\right)$$

$$p_j^{pret} = g_j^{col} p_j^{exp} + \left(1 - g_j^{col}\right) p_j^{unexp}$$

where p_j^{exp} represents risk of prematurity for pregnant women with GBS colonisation (exposed group) in country *j*; p_j^{unexp} represents risk for pregnant women who are not colonised by GBS; p_j^{pret} is the country-level risk of prematurity; and g_j^{col} represents prevalence of maternal GBS colonisation in country *j*.

Using the first approach, the excess number of preterm births estimated to be associated with GBS exposure was 596,600 (41,700-1,343,100), whilst using the second approach this quantity was 518,100 (36,900-1,142,300). The latter is presented in the *Results* section.

Computational methods

Bayesian analyses were performed using PyStan, the interface for Stan libraries in Python[24]; code is available upon request. Centered parameterisations were initially used for the hierarchical models; if divergences persisted after modifying the 'adapt_delta' parameter of the Hamiltonian Monte Carlo algorithm, non-centered parameterisations were used, as described by Betancourt and Girolami[25]. Figures and estimates were generated using 4,000 posterior samples from 4 chains.

Results Supplement

Figures

Figure S1. Incidence of early-infancy iGBS by region.

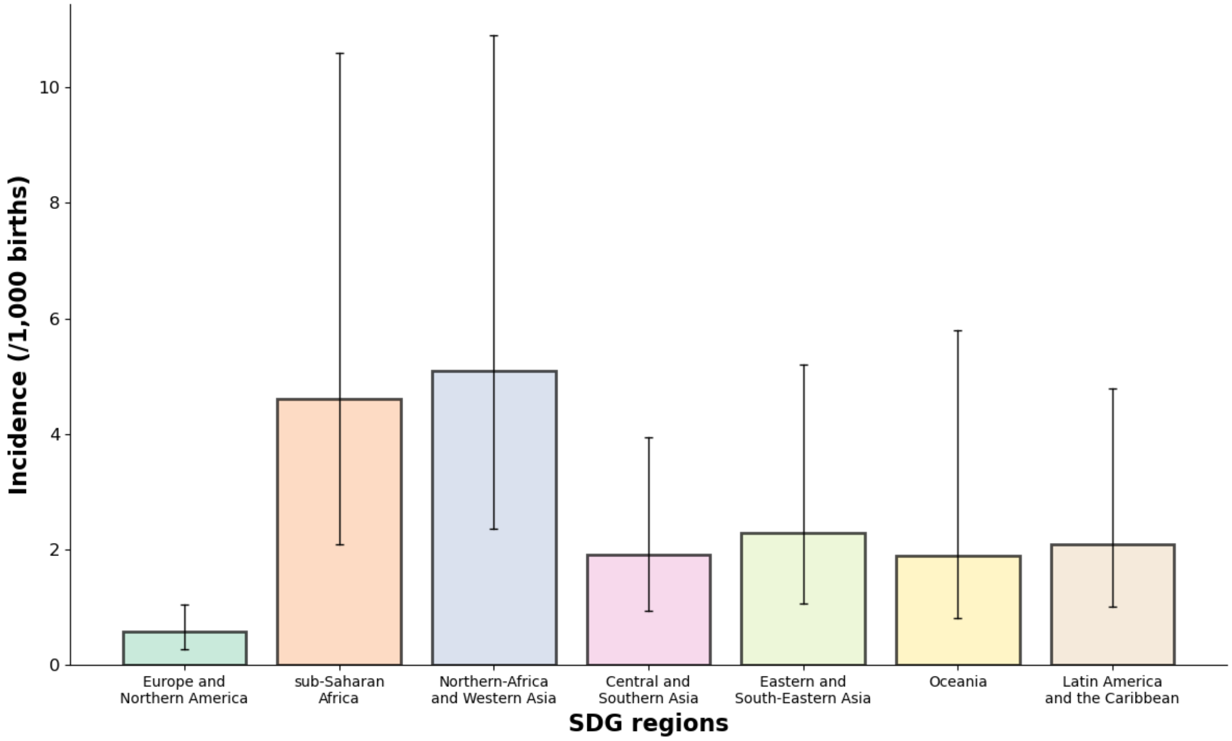

Figure S2. Proportion of stillbirths linked to GBS infection (x-axis). The y-axis indicates countries where studies were performed, or regional estimates (higher values of y-axis coordinates). For study-specific estimates, different shades of red correspond to 2.5 – 97.5, 25 – 75, and 40 – 60 percentile intervals; posterior medians are represented by vertical red lines and observed proportion in each study, by a black circle. Note that estimates for Oceania and Latin America regions were predicted from data available from other regions (green horizontal bar).

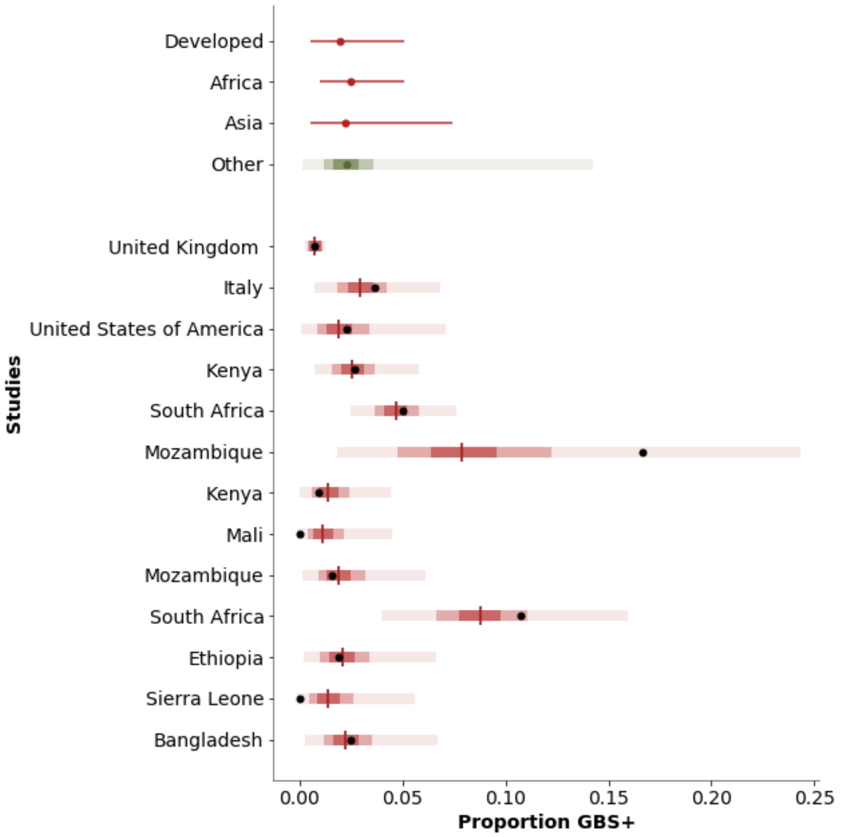

Figure S3. Proportions (x-axis) of iGBS cases diagnosed after the first week of life, i.e. LOGBS. Y-axis indicates countries where studies were performed, or regional estimates. Black circles represent observed proportions of iGBS cases in each study that were late-onset. Different shades of red correspond to 2.5 – 97.5, 25 – 75, and 40 – 60 percentile posterior intervals; posterior medians are represented by vertical red lines. The green bar represents the predicted proportion in Oceania, given studies in other regions.

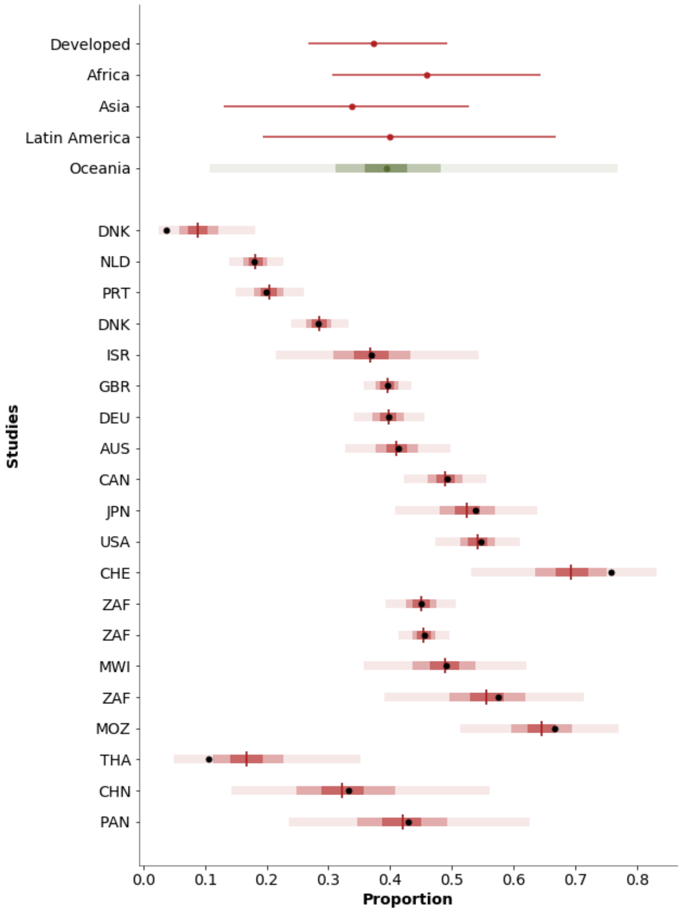

Figure S4. Posterior estimates of case fatality rates (x-axes) in EOGBS (panel A) and LOGBS (panel B) cases. Y-axes indicate countries (iso-3 codes) where studies were performed, or regional estimates. Black circles represent observed case fatality rates in each study. For study-specific estimates, different shades of red correspond to 2.5 – 97.5, 25 – 75, and 40 – 60 percentile intervals; posterior medians are represented by vertical red lines. The green bar represents the predicted CFR in Oceania, given studies in the other regions. Note that the x-axis range is not the same in the two panels.

A

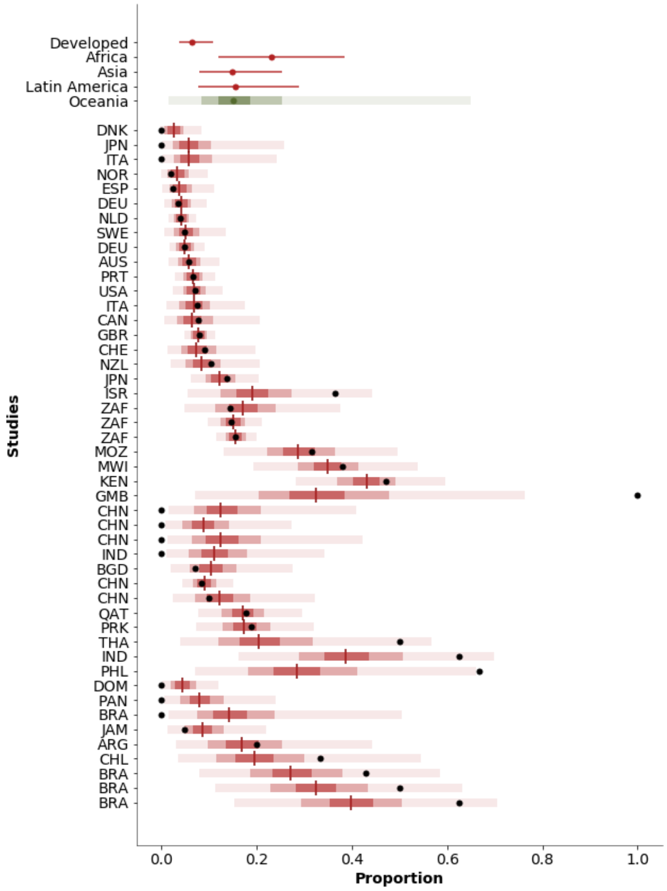

B

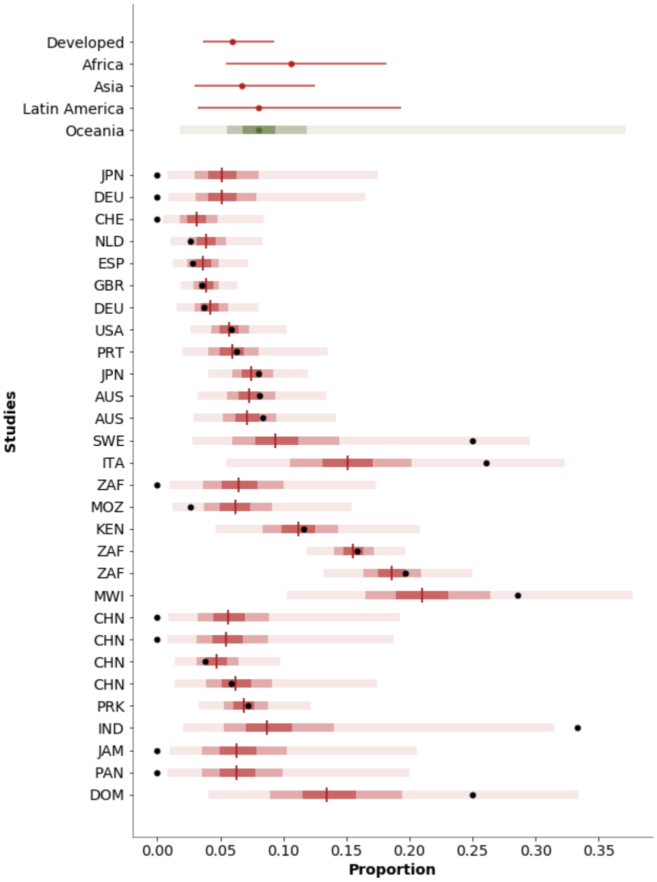
Developed
Africa
Asia
Latin America
Oceania
JPN
DEU
CHE
NLD
ESP
GBR
DEU
USA
PRT
JPN
AUS
AUS
SWE
ITA
ZAF
MOZ
KEN
ZAF
ZAF
MWI
CHN
CHN
CHN
CHN
PRK
IND
JAM
PAN
DOM
Studies
0.00
0.05
0.10
0.15
0.20
0.25
0.30
0.35
Proportion

Figure S5. Risk of moderate and severe NDI (panel A) or of NDI of any severity (panel B) after GBS meningitis.

A

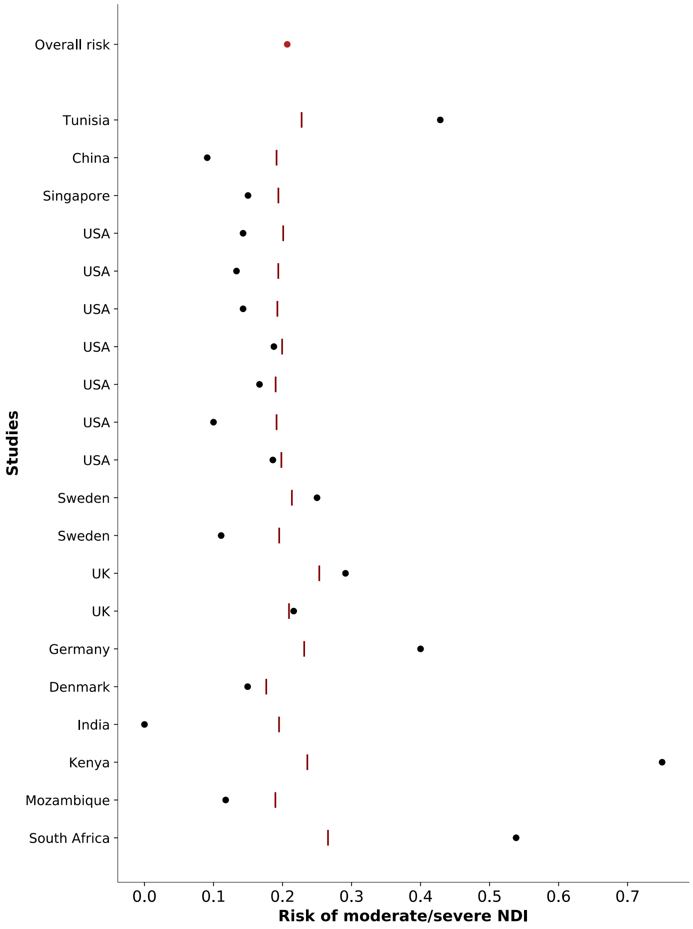

B

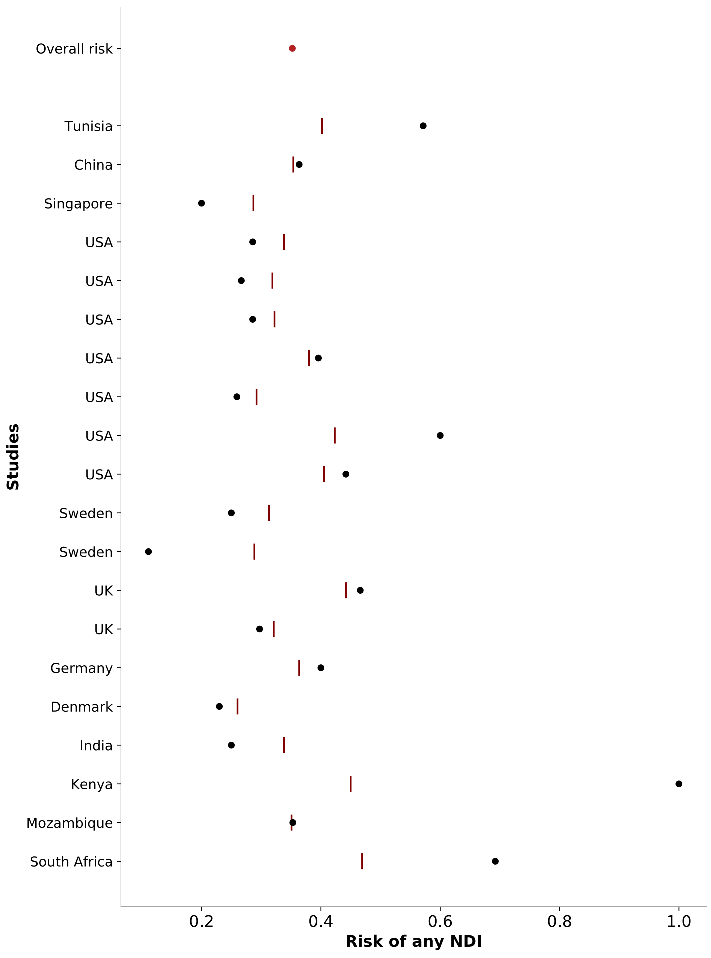
Overall risk
Tunisia
China
Singapore
USA
USA
USA
USA
USA
USA
USA
Sweden
Sweden
UK
UK
Germany
Denmark
India
Kenya
Mozambique
South Africa
Studies
0.2
0.4
0.6
0.8
1.0
Risk of any NDI

Figure S6. Risk of moderate and severe NDI or any severity NDI after GBS sepsis in low- and middle-income countries (LMIC; first two panels) and high-income countries (HIC; third and fourth panels).

LMIC

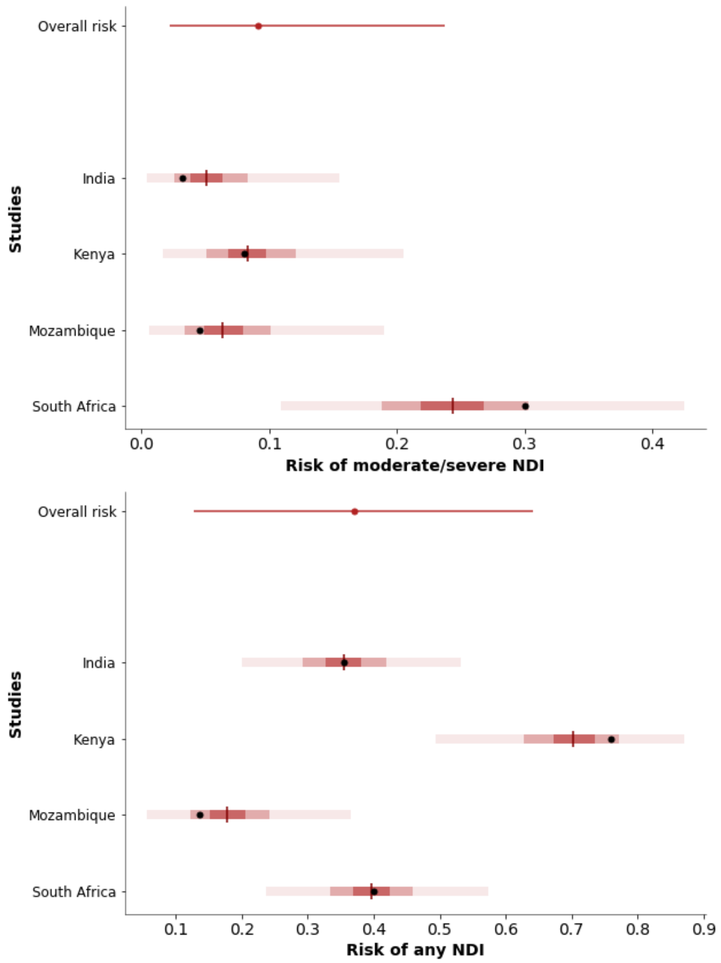

HIC

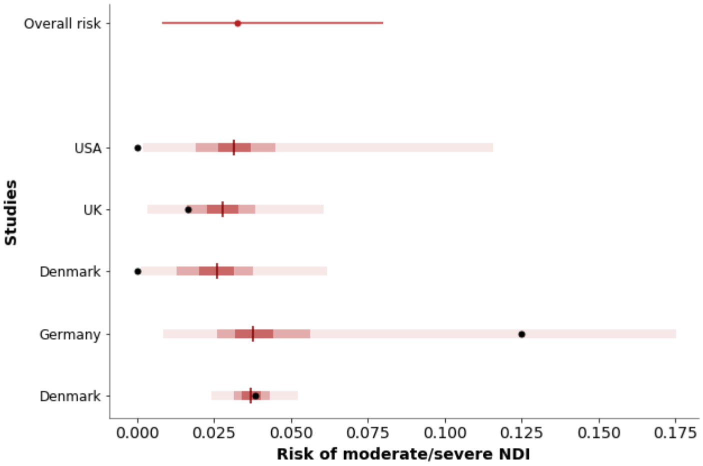

Overall risk
USA
UK
Denmark
Germany
Denmark
Studies
0.000
0.025
0.050
0.075
0.100
0.125
0.150
0.175
Risk of moderate/severe NDI

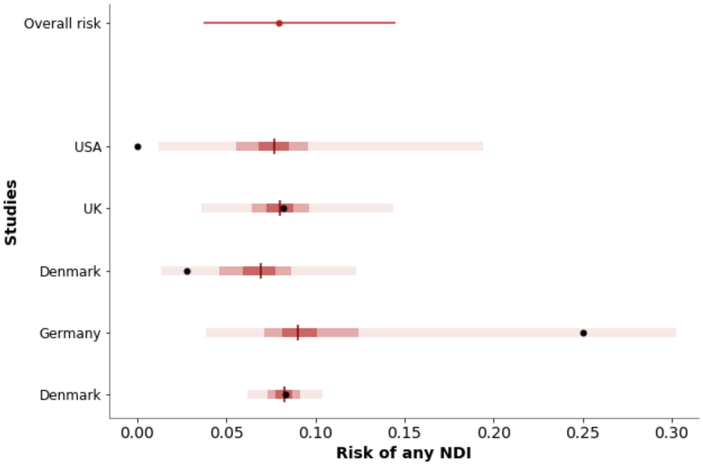

Overall risk
USA
UK
Denmark
Germany
Denmark
Studies
0.00
0.05
0.10
0.15
0.20
0.25
0.30
Risk of any NDI

Figure S7. Meta-analysis on the association between maternal GBS colonisation and preterm births. Data from case-control studies informed the prior distribution for the meta-analysis of cohort and cross-sectional studies, shown below. Black circles represent odds ratio in each study. Different shades of red correspond to 2.5 – 97.5, 25 – 75, and 40 – 60 percentile intervals; posterior medians are represented by vertical red lines.

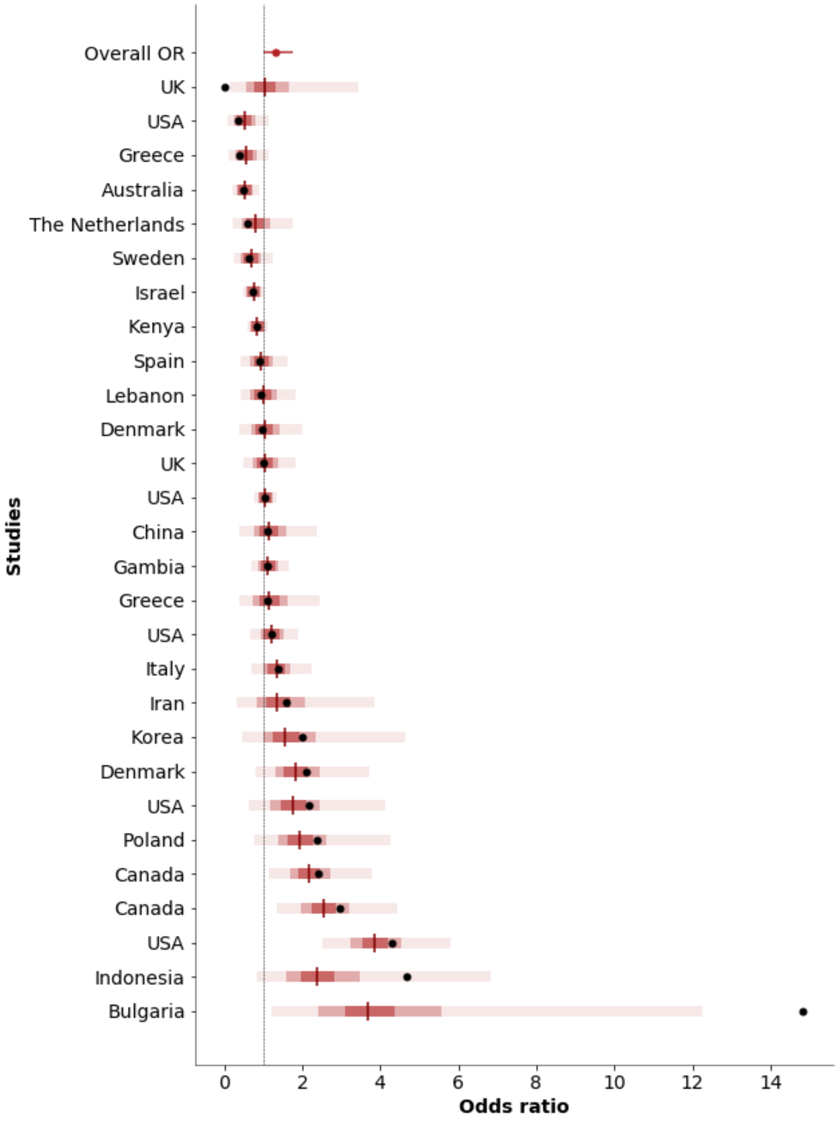

Figure S8. Estimates of the risk of maternal morbidity due to GBS infection.

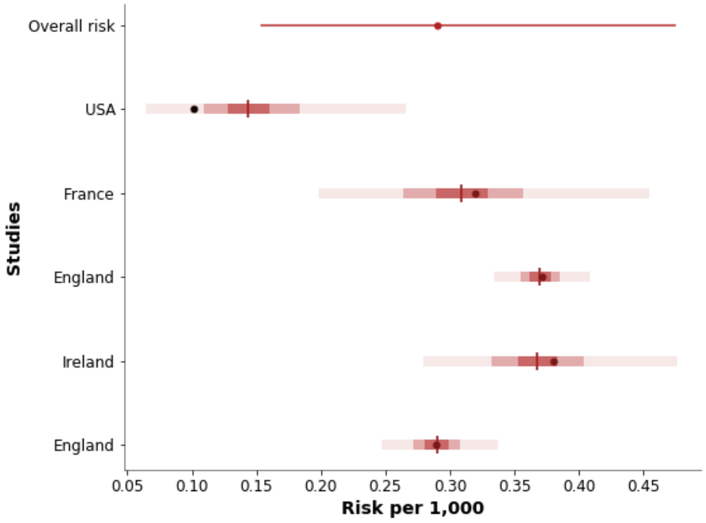

Tables

Table S1. Estimated numbers, in thousands, of GBS-colonised pregnant women and region-specific prevalences of GBS colonisation. We used country-specific numbers of births as weights to calculate global and region-specific prevalences. The last two digits of each number in the second column were rounded down.

SDG regions	GBS-colonised pregnancies (in thousands)	GBS colonisation prevalence (%)
Sub-Saharan Africa	6,000 (5,100 - 7,100)	16.1 (13.7 - 19.0)
Northern Africa and Western Asia	1,900 (1,600 - 2,400)	19.6 (16.2 - 24.0)
Central and Southern Asia	4,400 (3,700 - 5,200)	11.5 (9.6 - 13.6)
Eastern and South-Eastern Asia	3,100 (2,600 - 3,700)	10.4 (8.9 - 12.4)
Latin America and Caribbean	1,500 (1,300 - 1,700)	15.1 (13.3 - 17.1)
Oceania	120 (90 - 160)	18.6 (14.2 - 24.2)
Europe and Northern America	2,300 (2,000 - 2,700)	19.7 (17.3 – 22.6)
Global	19,700 (17,800 - 21,800)	14.1 (12.8 - 15.6)

Table S2. Posterior medians and 95% intervals of coefficients in the hierarchical model on maternal GBS colonisation.

	Median	95% interval
**Country-level covariates**		
Percent coverage of ATB for LRI	0.3	(0.07 - 0.53)
Maternal education	-0.18	(-0.48 - 0.11)
GNI	0.16	(0.03 - 0.29)
Neonatal mortality	0.22	(0.00 - 0.44)
HIV prevalence	0.19	(0.06 - 0.32)
Obesity prevalence	0.2	(0.08 - 0.31)
**Study-level covariates**		
Swab site	-0.23	(-0.39 - -0.07)
Culture method	-0.3	(-0.47 - -0.13)
**Standard deviation parameters**		
σ_c	0.33	(0.23 - 0.45)
σ_s	0.52	(0.47 - 0.58)

Table S3. Estimated numbers of EOGBS using a Bayesian evidence synthesis model that includes also data from incidence studies (see details in the *Supplementary Methods*).

**SDG regions**	**EOGBS**
Sub-Saharan Africa	85,100 (50,700 – 138,900)
Northern Africa and Western Asia	27,700 (16,300 – 45,400)
Central and Southern Asia	46,800 (29,300 – 74,700)
Eastern and South-Eastern Asia	42,800 (26,100 – 71,300)
Latin America and Caribbean	12,600 (8,000 – 19,500)
Oceania	700 (400 – 1,300)
Europe and Northern America	5,300 (3,300 – 8,100)
Global	222,500 (138,000 – 353,300)

Table S4. Bayesian estimates (posterior medians and 95% intervals) of region-specific proportions of iGBS cases diagnosed between days 7 and 89 (LOGBS).

Regions	Proportion of iGBS that are LOGBS (%)
Africa	46.1 (31.6 - 64.6)
Asia	34.1 (13.1 - 53.3)
Developed	37.4 (27.2 - 48.7)
Latin America and Caribbean	39.8 (20.0 - 66.6)
Oceania	39.8 (12.9 - 77.4)

Table S5. Region-specific case fatality rates (CFR), as proportions, in EOGBS and LOGBS cases. The wide uncertainty for Oceania reflects the fact that no studies from this region were included in the analysis.

**Regions**	**CFR – EOGBS**	**CFR – LOGBS**
Africa	0.23 (0.12 – 0.38)	0.10 (0.05 – 0.19)
Asia	0.15 (0.08 – 0.25)	0.07 (0.03 – 0.12)
Developed	0.06 (0.04 – 0.10)	0.06 (0.04 – 0.09)
Latin America and Caribbean	0.16 (0.08 – 0.28)	0.08 (0.03 – 0.20)
Oceania	0.15 (0.02 – 0.61)	0.08 (0.02 – 0.40)

Table S6. Estimated numbers of deaths in children who developed iGBS in 2020 by region. These estimates differ from estimates shown in **Table 2** in that here all children, regardless of access to skilled birth attendance, were assumed to have CFR presented in **Table S5**.

**SDG regions**	**Deaths**
Sub-Saharan Africa	29,000 (12,000 – 68,900)
Northern Africa and Western Asia	7,600 (3,200 – 17,100)
Central and Southern Asia	8,700 (3,700 – 20,000)
Eastern and South-Eastern Asia	8,300 (3,400 – 20,000)
Latin America and Caribbean	2,700 (1,100 – 6,800)
Oceania	150 (40 – 600)
Europe and Northern America	400 (100 – 800)
Global	58,300 (26,500 – 125,800)

Table S7. Region-specific percentages of stillbirths with evidence of GBS infection. Note that for Oceania and Latin America, no data were available, and we predicted percentages based on studies performed in other regions.

Region	Posterior Median (95% CI)
Developed	1.9 (0.6 – 4.8) %
Africa	2.5 (1.1 – 4.9) %
Asia	2.2 (0.5 – 7.8) %
Oceania/Latin America	2.2 (0.4 – 14.1) %

Table S8. Sensitivity analyses under different prior assumptions. For each analysis, although we assessed changes in summaries of most parameters, we selected a few relevant parameters or quantities to be presented. Note that here, different from equations presented in the *Supplementary Methods*, the normal distribution is parameterised with the scale parameter, rather than the variance, to follow *Stan* software usage. Abbreviations: Pr_S-Af, percentage of stillbirths associated with GBS in Sub-Saharan Africa; Pr_New, predicted percentage of stillbirths associated with GBS in regions with no data; Pr_LO, proportion of all invasive GBS cases that are LOGBS; CFR_EO and CFR_LO, overall CFRs for EOGBS and LOGBS; CFR_EO_new and CFR_LO_new, predicted CFRs in a region with no study; NDI_ms_sepsis, risk of moderate/severe NDI after GBS sepsis; NDI_ms_meningitis, risk of moderate/severe NDI after GBS meningitis; Mat_risk, risk of GBS-related maternal disease per 1,000 deliveries.

Model	Prior Assumption	Primary analysis	Sensitivity analysis
Stillbirth	$\mu_{g-stil} \sim Normal(0,5)$<br>$\sigma_{g-stil} \sim Normal(0,1)$	Pr_S-Af = 2.5 (1.1 – 4.9)<br>Pr_New = 2.2 (0.4 – 14.1)	Pr_S-Af = 2.4 (1.1 – 5.1)<br>Pr_New = 2.2 (0.3 – 13.6)
Stillbirth	$\mu_{g-stil} \sim Normal(0,5)$<br>$\sigma_{g-stil} \sim Normal(0,5)$	Pr_S-Af = 2.5 (1.1 – 4.9)<br>Pr_New = 2.2 (0.4 – 14.1)	Pr_S-Af = 2.6 (1.0 – 5.1)<br>Pr_New = 2.2 (0.7 – 53.4)
Stillbirth	$\mu_{g-stil} \sim Normal(0,5)$<br>$\sigma_{g-stil} \sim Exponential(1)$	Pr_S-Af = 2.5 (1.1 – 4.9)<br>Pr_New = 2.2 (0.4 – 14.1)	Pr_S-Af = 2.4 (1.0 – 4.9)<br>Pr_New = 2.2 (0.3 – 11.8)
LOGBS	$\mu_{g-lo} \sim Normal(0,5)$<br>$\sigma_{g-lo} \sim Normal(0,1)$	Pr_LO = 0.40 (0.23 – 0.57)	Pr_LO = 0.39 (0.20 – 0.60)
LOGBS	$\mu_{g-lo} \sim Normal(0,5)$<br>$\sigma_{g-lo} \sim Normal(0,5)$	Pr_LO = 0.40 (0.23 – 0.57)	Pr_LO = 0.39 (0.15 – 0.65)
EO-CFR	$\mu_{g-cfr} \sim Normal(0,5)$<br>$\sigma_{g-cfr} \sim Normal(0,1)$	CFR_EO = 0.14 (0.07 – 0.32)<br>CFR_EO_New = 0.14 (0.02 – 0.61)	CFR_EO = 0.13 (0.05 – 0.31)<br>CFR_EO_New = 0.13 (0.02 – 0.58)
EO-CFR	$\mu_{g-cfr} \sim Normal(0,5)$<br>$\sigma_{g-cfr} \sim Normal(0,5)$	CFR_EO = 0.14 (0.07 – 0.32)<br>CFR_EO_New = 0.14 (0.02 – 0.61)	CFR_EO = 0.14 (0.03 – 0.47)<br>CFR_EO_New = 0.13 (0.00 – 0.84)
LO-CFR	$\mu_{g-cfr} \sim Normal(0,5)$<br>$\sigma_{g-cfr} \sim Normal(0,1)$	CFR_LO = 0.08 (0.05 – 0.19)<br>CFR_LO_New = 0.08 (0.02 – 0.38)	CFR_LO = 0.07 (0.03 – 0.15)<br>CFR_LO_New = 0.07 (0.01 – 0.28)
LO-CFR	$\mu_{g-cfr} \sim Normal(0,5)$<br>$\sigma_{g-cfr} \sim Normal(0,5)$	CFR_LO = 0.08 (0.05 – 0.19)<br>CFR_LO_New = 0.08 (0.02 – 0.38)	CFR_LO = 0.07 (0.02 – 0.24)<br>CFR_LO_New = 0.07 (0.01 – 0.49)
NDI sepsis (HIC)	$\mu^{NDI}_{g-syn} \sim Normal(0,5)$<br>$\sigma_{g-syn} \sim Normal(0,5)$	NDI_ms_Sepsis = 0.03 (0.01 – 0.07)	NDI_ms_Sepsis = 0.03 (0.01 – 0.11)
NDI sepsis (LMIC)	$\mu^{NDI}_{g-syn} \sim Normal(0,5)$<br>$\sigma_{g-syn} \sim Normal(0,1)$	NDI_ms_Sepsis = 0.09 (0.02 – 0.23)	NDI_ms_Sepsis = 0.09 (0.02 – 0.26)
NDI sepsis (LMIC)	$\mu^{NDI}_{g-syn} \sim Normal(0,5)$<br>$\sigma_{g-syn} \sim Normal(0,5)$	NDI_ms_Sepsis = 0.09 (0.02 – 0.23)	NDI_ms_Sepsis = 0.09 (0.01 – 0.51)
NDI meningitis	$\mu^{NDI}_{g-syn} \sim Normal(0,5)$<br>$\sigma_{g-syn} \sim Normal(0,5)$	NDI_ms_meningitis = 0.21 (0.16 – 0.26)	NDI_ms_meningitis = 0.20 (0.15– 0.26)
Maternal morbidity	$\mu_{g-mat} \sim Normal(0,10)$<br>$\sigma_{g-mat} \sim Normal(0,1)$	Mat_risk = 0.29 (0.16 – 0.53)	Mat_risk = 0.28 (0.15 – 0.52)
Maternal morbidity	$\mu_{g-mat} \sim Normal(0,10)$<br>$\sigma_{g-mat} \sim Normal(0,10)$	Mat_risk = 0.29 (0.16 – 0.53)	Mat_risk = 0.29 (0.13 – 0.67)

Table S9. Limitations and assumptions

Outcome	Limitations and assumptions
GBS colonisation during pregnancy	- Majority of studies used culture methods; PCR methods are likely more sensitive;<br>- Within-country geographical variation not modelled;
Early-onset iGBS risk (Approach I)	- High heterogeneity in risks in individual studies, possibly due to differences in design, diagnostics and study population;<br>- Only culture-confirmed EOGBS considered, which might lead to under-estimation depending on culture sensitivity;<br>- Most studies from high income countries;<br>- By not differentiating between microbiology screening- and risk factors-based IAP, which might be less effective in reducing EOGBS [26], we might have underestimated the number of EOGBS cases where the latter approach is used;
Early-onset iGBS risk (Approach II)	- Although this evidence synthesis model partially accounts for the likely under-reporting/under-ascertainment in studies directly estimating GBS incidence, additional, context-specific information on degree of under-reporting and under-ascertainment would improve estimation;
Late-onset iGBS risk	- An assumption in modelling this outcome is that the under-estimation of EOGBS and LOGBS case numbers are similar; however it is not implausible that under-reporting is higher in EOGBS (e.g. due to home delivery) or in LOGBS (e.g. limited capacity or hesitation for cerebrospinal fluid sampling);
Case fatality rates (EOGBS and LOGBS)	- Several studies with small number of cases (publication bias?);<br>- Possible bias due to case ascertainment in some settings, as suggested in [27] ;
Neurodevelopmental impairment after GBS meningitis	- Limited data outside the USA and Europe;<br>- Variation in methods of NDI assessment and case capture between studies;<br>- Absence of an unexposed (children with no history of iGBS) group in many of these studies;
Neurodevelopmental impairment after GBS sepsis	- Variation in risk observed between studies;<br>- For both sepsis and meningitis calculations, it is difficult to define a counterfactual risk that could be reasonably applied globally when estimating excess number of cases;
Stillbirths	- Limited data from Asia;
Maternal morbidity linked to GBS	- All studies from high-income countries;<br>- Not all maternal infections caused by GBS are captured by definitions.
Preterm births associated with maternal GBS colonisation	- Considerable variation in study design, including timing of exposure assessment and study population (e.g. exclusion or not of women with recent antibiotic use)

Table S10. Posterior median and 95% interval of scale parameters, which capture between study variability and for some of the outcomes between region variability (see Supplementary Methods for details)

Parameter	Median (95% interval)
σ_{g-lo}	0.45 (0.03 – 1.43)
σ_{s-lo}	0.80 (0.55 – 1.23)
σ_{g-cfr} (EO)	0.75 (0.27 – 1.76)
σ_{s-cfr} (EO)	0.87 (0.54 – 1.30)
σ_{g-cfr} (LO)	0.49 (0.04 – 1.53)
σ_{s-cfr} (LO)	0.68 (0.30 – 1.17)
σ_{g-syn} (Meningitis)	0.33 (0.03 – 0.82)
σ_{g-syn} (HIC-sepsis)	0.53 (0.02 – 1.85)
σ_{g-syn} (LMIC-sepsis)	0.96 (0.17 – 2.04)
σ_{g-stil}	0.43 (0.02 – 1.69)
σ_{s-stil}	0.95 (0.50 – 1.73)
σ_{g-mat}	0.49 (0.16 – 1.37)

References

1. Goncalves BP, Procter SR, Clifford S, et al. Estimation of country-level incidence of early-onset invasive Group B Streptococcus disease in infants using Bayesian methods. *PLoS Comput Biol* 2021; **17**(6): e1009001.
2. Russell NJ, Seale AC, O'Driscoll M, et al. Maternal Colonization With Group B Streptococcus and Serotype Distribution Worldwide: Systematic Review and Meta-analyses. *Clin Infect Dis* 2017; **65**(suppl_2): S100-S11.
3. Seale AC, Bianchi-Jassir F, Russell NJ, et al. Estimates of the Burden of Group B Streptococcal Disease Worldwide for Pregnant Women, Stillbirths, and Children. *Clin Infect Dis* 2017; **65**(suppl_2): S200-S19.
4. Russell NJ, Seale AC, O'Sullivan C, et al. Risk of Early-Onset Neonatal Group B Streptococcal Disease With Maternal Colonization Worldwide: Systematic Review and Meta-analyses. *Clin Infect Dis* 2017; **65**(suppl_2): S152-S9.
5. Benitz WE, Gould JB, Druzin ML. Risk factors for early-onset group B streptococcal sepsis: estimation of odds ratios by critical literature review. *Pediatrics* 1999; **103**(6): e77.
6. Melin P, Efstratiou A. Group B streptococcal epidemiology and vaccine needs in developed countries. *Vaccine* 2013; **31 Suppl 4**: D31-42.
7. Le Doare K, O'Driscoll M, Turner K, et al. Intrapartum Antibiotic Chemoprophylaxis Policies for the Prevention of Group B Streptococcal Disease Worldwide: Systematic Review. *Clin Infect Dis* 2017; **65**(suppl_2): S143-S51.
8. Madrid L, Seale AC, Kohli-Lynch M, et al. Infant Group B Streptococcal Disease Incidence and Serotypes Worldwide: Systematic Review and Meta-analyses. *Clin Infect Dis* 2017; **65**(suppl_2): S160-S72.
9. Luck S, Torny M, d'Agapeyeff K, et al. Estimated early-onset group B streptococcal neonatal disease. *Lancet* 2003; **361**(9373): 1953-4.
10. Dagnew AF, Cunnington MC, Dube Q, et al. Variation in reported neonatal group B streptococcal disease incidence in developing countries. *Clin Infect Dis* 2012; **55**(1): 91-102.
11. Berardi A, Lugli L, Baronciani D, et al. Group B streptococcal infections in a northern region of Italy. *Pediatrics* 2007; **120**(3): e487-93.
12. Kohli-Lynch M, Russell NJ, Seale AC, et al. Neurodevelopmental Impairment in Children After Group B Streptococcal Disease Worldwide: Systematic Review and Meta-analyses. *Clin Infect Dis* 2017; **65**(suppl_2): S190-S9.
13. Horvath-Puho E, van Kassel MN, Goncalves BP, et al. Mortality, neurodevelopmental impairments, and economic outcomes after invasive group B streptococcal disease in early infancy in Denmark and the Netherlands: a national matched cohort study. *Lancet Child Adolesc Health* 2021.
14. Paul P, Procter SR, Dangor Z, et al. Quantifying long-term health and economic outcomes for survivors of group B Streptococcus invasive disease in infancy: protocol of a multi-country study in Argentina, India, Kenya, Mozambique and South Africa. *Gates Open Res* 2020; **4**(138).
15. Blencowe H, Vos T, Lee AC, et al. Estimates of neonatal morbidities and disabilities at regional and global levels for 2010: introduction, methods overview, and relevant findings from the Global Burden of Disease study. *Pediatr Res* 2013; **74 Suppl 1**: 4-16.

16. Seale AC, Blencowe H, Zaidi A, et al. Neonatal severe bacterial infection impairment estimates in South Asia, sub-Saharan Africa, and Latin America for 2010. *Pediatr Res* 2013; **74 Suppl 1**: 73-85.
17. Seale AC, Blencowe H, Bianchi-Jassir F, et al. Stillbirth With Group B Streptococcus Disease Worldwide: Systematic Review and Meta-analyses. *Clin Infect Dis* 2017; **65**(suppl_2): S125-S32.
18. Taylor AW, Blau DM, Bassat Q, et al. Initial findings from a novel population-based child mortality surveillance approach: a descriptive study. *Lancet Glob Health* 2020; **8**(7): e909-e19.
19. https://www.who.int/data/gho (Global Health Observatory data repository). 2020).
20. Hall J, Adams NH, Bartlett L, et al. Maternal Disease With Group B Streptococcus and Serotype Distribution Worldwide: Systematic Review and Meta-analyses. *Clin Infect Dis* 2017; **65**(suppl_2): S112-S24.
21. Collin SM, Shetty N, Lamagni T. Invasive Group B Streptococcus Infections in Adults, England, 2015-2016. *Emerg Infect Dis* 2020; **26**(6): 1174-81.
22. Bianchi-Jassir F, Seale AC, Kohli-Lynch M, et al. Preterm Birth Associated With Group B Streptococcus Maternal Colonization Worldwide: Systematic Review and Meta-analyses. *Clin Infect Dis* 2017; **65**(suppl_2): S133-S42.
23. Chawanpaiboon S, Vogel JP, Moller AB, et al. Global, regional, and national estimates of levels of preterm birth in 2014: a systematic review and modelling analysis. *Lancet Glob Health* 2019; **7**(1): e37-e46.
24. Stan User's Guide (Version 2.24): Stan Development Team.
25. Betancourt M, Girolami M. Hamiltonian Monte Carlo for Hierarchical Models. Current Trends in Bayesian Methodology with Applications. New York: Chapman and Hall/CRC; 2015.
26. Schrag SJ, Zell ER, Lynfield R, et al. A population-based comparison of strategies to prevent early-onset group B streptococcal disease in neonates. *N Engl J Med* 2002; **347**(4): 233-9.
27. Gray KJ, Bennett SL, French N, Phiri AJ, Graham SM. Invasive group B streptococcal infection in infants, Malawi. *Emerg Infect Dis* 2007; **13**(2): 223-9.
